# HSP60 controls mitochondrial ATP generation for optimal virus-specific IL-21-producing CD4 and cytotoxic CD8 memory T cell responses

**DOI:** 10.1038/s42003-024-07326-8

**Published:** 2024-12-21

**Authors:** Nazanin Ghahari, Saina Shegefti, Mahsa Alaei, Amine Amara, Roman Telittchenko, Stéphane Isnard, Jean-Pierre Routy, David Olagnier, Julien van Grevenynghe

**Affiliations:** 1 Institut national de la recherche scientifique (INRS)-Centre Armand-Frappier Santé Biotechnologie, 531 boulevard des Prairies, H7V 1M7 Laval, QC Canada; 2 Chronic Viral Illness Service and Division of Hematology, McGill University Health Centre, Glen site, H4A 3J1 Montreal, Quebec Canada; 3 Aarhus University; Department of Biomedicine, Aarhus C, 8000 Denmark

**Keywords:** T-cell receptor, Lymphocyte activation

## Abstract

We have shown that virus-specific CD4 and CD8 memory T cells (TM) induce autophagy after T cell receptor (TCR) engagement to provide free glutamine and fatty acids, including in people living with HIV-1 (PLWH). These nutrients fuel mitochondrial ATP generation through glutaminolysis and fatty acid oxidation (FAO) pathways, to fulfill the bioenergetic demands for optimal IL-21 and cytotoxic molecule production in CD4 and CD8 cells, respectively. Here, we expand our knowledge on how the metabolic events that occur in the mitochondria of virus-specific TM down-stream of the autophagy are regulated. We show that HSP60 chaperone positively regulates the protein levels for multiple glutaminolysis- and FAO-related enzymes, thereby actively fueling the levels of cellular alpha-ketoglutarate (αKG) and related mitochondrial ATP-dependent antiviral T cell immunity in both CD4 and CD8 TM. Finally, we provide a way to rescue defective ATP generation in mitochondria and dependent effector functions in virus-specific TM including anti-HIV-1 protective responses, when HSP60 expression is impaired after TCR engagement in patients, in the form of dimethyl 2-oxoglutarate (DMKG) supplementation.

## Abstract (web summary)

Antiviral immune function in both CD4 and CD8 memory T cells, which relies on effective ATP generation in mitochondria, involves the resident chaperone HSP60 by assisting the expression of multiples mitochondrial enzymes after cell activation

## Introduction

In recent years, the integration of metabolism within immunity, known as “immuno-metabolism”, has been at the forefront of T cell immunology research, primarily focusing on the metabolic rewiring that follows TCR engagement with pathogen-specific antigens (Ags)^1–4^. It is now well-acknowledged that, in a few minutes after TCR engagement by viral Ags, both CD4 and CD8 TM increase glucose uptake and perform aerobic glycolysis; a metabolic state that is usually adopted by highly proliferative cells like cancer cells in which, glucose is fermented into lactate rather than be oxidized in mitochondria^5–8^. This rapid induction of aerobic glycolysis enables effector TM to fulfill bioenergetic demands that are requested not only for cell survival and growth, but also for their production of pro-inflammatory interferon gamma (IFN-γ), tumor necrosis factor alpha (TNF-α), and interleukin 2 (IL-2) cytokines^7,9,10^. However, the metabolic rewiring that is induced in virus-specific CD4 and CD8 T cells after TCR engagement is not restricted to aerobic glycolysis, but also includes the use of mitochondrial oxidative phosphorylation (OXPHOS) respiratory system in a later phase of activation. Indeed, both activated CD4 and CD8 TM have higher spare respiratory capacity (SRC) and adenosine triphosphate (ATP)-linked respiration when compared to non-activated cells starting at 6 hours post-TCR engagement and which is still detectable after 72 hours of cell culture^11–15^.

The levels of mitochondrial ATP that are generated by OXPHOS in virus-specific TM greatly influences protective immunity by bolstering synergic effector functions such as IL-21 production in CD4 T follicular helper (Tfh) cells and Perforin/Granzyme-B dual-expression in cytotoxic CD8 T lymphocytes (CTL). It is particularly the case during chronic human immunodeficiency virus type 1 (HIV-1) in which virus-protective T-cell immunity strongly depends on optimal ATP production in mitochondria^11,13,14,16^. In fact, the vast majority of PLWH, despite long-lasting administration of antiretroviral therapy (ART), elicits defective OXPHOS-driven ATP generation after TCR engagement including with HIV-1 peptide pools^13,14,16^. This results in lower IL-21 and cytotoxic molecule production when compared to both HIV-1-uninfected control group and elite controllers (ECs) who naturally control HIV-1 infection for years without ART^17–20^. Importantly, effective OXPHOS-driven ATP generation in virus-specific CD4 and CD8 TM is not only critical for ensuring strong immune protection against HIV-1, but represents a common requirement for other viruses, such as adenovirus, cytomegalovirus (CMV), Epstein-Barr virus (EBV) and influenza virus^13,14^.

Mechanistically speaking, our previous works have provided critical insights on how virus-specific TM regulate mitochondrial ATP-dependent IL-21, Perforin, and Granzyme-B expressions. After TCR engagement, both virus-specific CD4 and CD8 TM induce autophagy, a catabolic process by which they can degrade and recycle cellular proteins and lipids to provide endogenous glutamine and fatty acids^13,14,16,21^. Once produced, glutamine and fatty acids enter the mitochondria where they are then transformed by a series of enzymatic reactions, known as glutaminolysis and FAO, respectively, into oxidized intermediates. These intermediates can further be integrated into the tricarboxylic acid (TCA) cycle to assist the OXPHOS-driven ATP generation^22^. Our results showed that virus-specific CD4 TM use glutaminolysis to meet energy demands for IL-21 production^13^, while the vFAO pathway is specifically requested for virus-specific CTL immunity^14^. In fact, inhibiting glutaminolysis in CD4 TM with BPTES and R162, or FAO pathway in CD8 TM with etomoxir (Eto) fully prevented mitochondrial ATP generation after TCR engagement, resulting in low levels that were like non-activated cells^13,14^. Finally, although we confirmed that inducing autophagy in ART-treated PLWH with AICAR drug was effective in restoring high IL-21 and cytotoxic molecule production in the same range than ECs and uninfected control group, it was nonetheless ineffective in two of them. For these reasons, we felt it was mandatory to strengthen our knowledge on how mitochondria of virus-specific TM further regulate the glutaminolytic and FAO pathways for optimal ATP generation and better rescue strategy.

Heat shock protein 60 (HSP60), which mainly resides in mitochondria, belongs to a conserved family of molecular chaperones that are mainly transactivated by the heat shoch factor 1 (HSF1)^23,24^. These chaperones are known to improve the expression of various proteins by assisting their folding into a stable and functional conformation^25,26^. Interestingly, a recent survey of mitochondrial HSP60 interactors includes multiple enzymes related to both glutaminolysis and FAO, and the lipid transporter carnitine palmitoyltransferase II (CPT2)^27^. In fact, there is evidence that specific *Hspd1* gene silencing [HSP60 inhibition] in cancer cells not only leads to reduced protein levels for the FAO-related short-chain enoyl-CoA hydratase 1 (ECHS1), hydroxy acyl-CoA (HADH) and middle-chain acyl-CoA dehydrogenase MCAD enzymes^28–30^, but also results in defective mitochondrial ATP-linked respiration^31^. However, if and how HSP60 regulates the mitochondrial energy metabolism of virus-specific TM has not yet been investigated.

Therefore, we decided to assess the impact of HSP60 protein inhibition in activated CD4 and CD8 TM, including virus-specific cells, by using specific *Hspd1* gene silencing and cell treatment with the selective HSF1 inhibitor KRIB11 (KR). First, our analysis revealed that the expression of mitochondrial HSP60 increased in virus-specific CD4 and CD8 TM after TCR engagement in an HSF1-dependent manner. HSP60 inhibition in CD4 and CD8 TM led to lower protein levels for several glutaminolytic and FAO-related enzymes after TCR engagement, along with reduced levels for the rate-determining TCA cycle intermediate alpha-ketoglutarate (αKG) and mitochondrial ATP-linked respiration. Accordingly, HSP60 inhibition in virus-specific TM resulted in defective mitochondrial ATP-dependent IL-21 production in CXCR5^+^CXCR3^neg^ Tfh cells [cell subset representing more than 80% of IL-21-producing CD4 TM] and, Perforin and Granzyme-B dual-expression in CTL, neither influencing autophagy nor glycolysis-dependent production of IFN-γ, TNF-α and IL-2 cytokines. DMKG, which is a diester and cell-permeable analog of αKG^32^, supplemented reduced αKG levels both in activated CD4 and CD8 TM, thereby rescuing their mitochondrial ATP-dependent effector immunity despite glutaminolysis [CD4], FAO [CD8] and HSP60 inhibition. To summarize, we provide evidence that mitochondrial HSP60 is a positive regulator of ATP generation in both virus-specific CD4 and CD8 TM and must be considered for proper virus-specific immune protection.

## Results

### HSP60 expression increases in both CD4 and CD8 TM, including virus-specific cells, in an HSF1-dependent manner after TCR engagement

Evidence shows that the activity of HSF1, the main factor that transactivate the expression of the heat shock protein (HSP) family members^23,24^, is quickly induced in T cells after TCR engagement^33^. As it is still poorly understood, we decided to assess if and how intracellular HSP60 protein is regulated within CD4 and CD8 T cells during T cell activation, including in their virus-specific CD45RA^neg^memory T cells (TM).

**Fig. 1:**
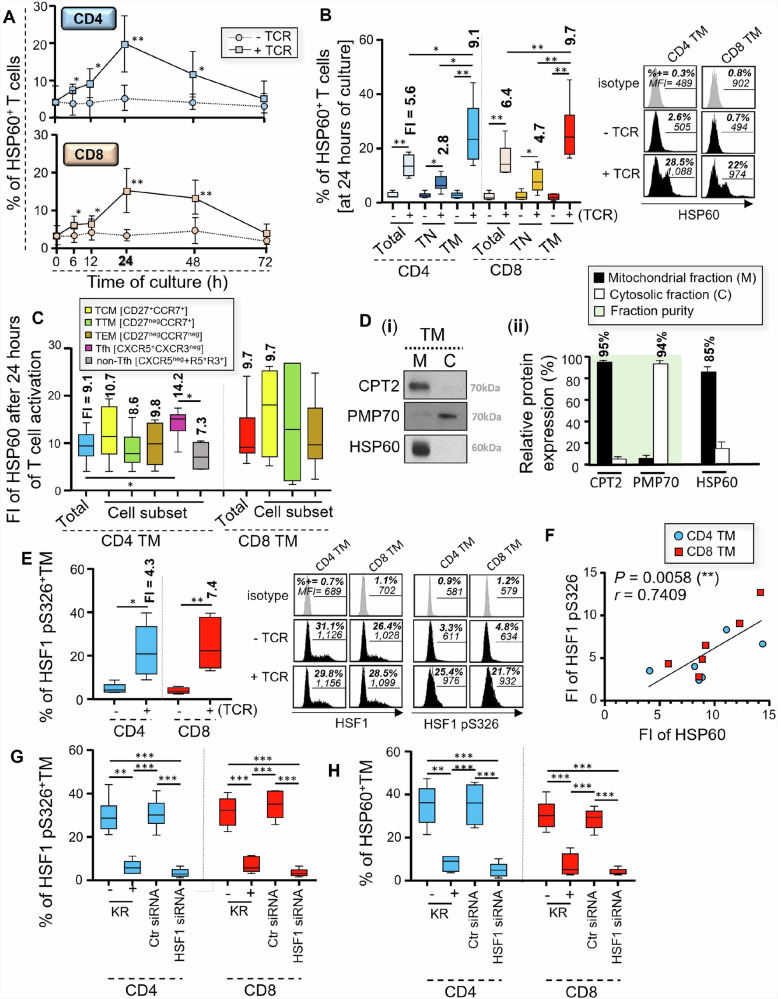
Mitochondrial HSP60 expression increases in CD4 and CD8 TM after TCR engagement in an HSF1-dependent manner. Briefly, we either activated PBMC with anti-CD3/CD28 Abs or we did not. **A** Cells were then collected at 6, 12, 24, 48 and 72 hours of culture to assess the percentages (%) of HSP60^+^ CD4 and CD8 T cells. **B** % of HSP60 positivity in total, CD45RA^+^ naïve (TN), and CD45RA^neg^ memory (TM) CD4 and CD8 T cells at 24 hours of culture [selected for maximum HSP_60_ expression in activated TM]. Fold increases (FI) of HSP60 expression in T cells after TCR engagement are also indicated in bold. FI = [% of HSP60 positivity in activated T cells] / [% of HSP60 positivity in non-activated T cells]. **C** FI of HSP60 expression in different CD4 and CD8 TM subsets at 24 hours post-T cell activation with quantitative values indicated in bold. **D** Protein levels of mitochondrial CPT2, cytosolic PMP70, and HSP60 in mitochondria (M) and cytosol fractions (C) in activated TM. Data shown are (**i**) the representative blots and **(ii)** relative protein levels calculated with Image J software. Protein % of the overall pool within M or C are also indicated in bold. **E** % of active HSF1 pS326^+^ CD4 and CD8 TM at 24 hours of culture [after normalization with total HSF1 expression]. FI of HSF1 pS326 expression in T cells after TCR engagement are also indicated in bold. **F** Correlation between FI of HSF1 pS326 and HSP60 expression at 24 hours post-activation. N = 12 TM. **G** % of HSF1 pS326^+^ and **H** HSP60^+^ CD4 and CD8 TM at 24 hours post-activation, w/wo specific *Hsf1* gene silencing and KR. **A**–**E**, **G**, **H** Results represent the mean relative ± SD of *n* = 6, except for **D** with *n* = 3. Of note, representative black histograms of HSP60, HSF1 (total and active pS326 forms), including their respective grey isotype controls are also shown for CD4 and CD8 TM at 24 hours of culture w/wo TCR engagement.

First, peripheral blood mononuclear cells (PBMC) were TCR-engaged with anti-CD3 and anti-CD28 antibodies (Abs) for 6 to 72 hours. We then collected the cells to assess the time-dependent intracellular expression of HSP60 in CD4 and CD8 T cells after TCR engagement. Our data show that intracellular HSP60 expression increased in both CD4 and CD8 T cells, starting at 6 hours post-activation, but reached its maximum at 24 hours post-activation whilst remaining consistently low in non-activated cells (Fig. 1A). Nevertheless, HSP60 expression in activated T cells started to decrease after 48 hours of culture and was comparable to non-activated cells at 72 hours of culture^34^. HSP60 expression was increased to the maximum at 24 hours post-activation in the overall T cell population, but the strongest induction of HSP60 was always observed in TM when compared to CD45RA^+^ naïve T cells (TN) for both CD4 and CD8 cells [Fold increase; FI = 9.1 and 9.7 for TM, and 2.8 and 4.7 for TN, respectively in CD4 and CD8 T cells] (Fig. 1B and Supplementary Fig. 1A). Therefore, we further assessed the levels of HSP60 increase after 24 hours of TCR engagement in distinct TM subsets of CD4 and CD8 T cells, including the long-lasting central (TCM), transitional (TTM) and effector memory (TEM) T cells that are defined by differential expression of CD27 and CCR7 markers^13,35–38^. We further included the analysis of peripheral CD45RA^neg^ CD4^+^CXCR5^+^ CXCR3^neg^ T follicular helper (Tfh) cells, since they produce large amounts of IL-21 cytokine to sustain both CD8 and B cell immunity during viral infections such as HIV-1^19,39,40^. Although we found similar fold increases (FI) of HSP60 between TCM, TTM and TEM for both CD4 and CD8 at 24 hours post-activation, Tfh cells displayed stronger HSP60 induction after TCR engagement [FI = 14.2] when compared to CXCR5^neg^ and CXCR5^+^CXCR3^+^ non-Tfh cells [FI = 7.3] (Fig. 1C). Since HSP60 has been reported to be mainly a mitochondrial chaperone^25,41^, we decided to assess if it was also the case in activated TM. At 24 hours of T cell activation, TM were collected and lysed to isolate their mitochondrial and cytosolic fractions, whose purity superior to 94% was respectively validated by western blotting with the mitochondrial CPT2 lipid transporter and peroximosal membrane protein 70 (PMP70). Our data confirmed that more than 85% of the overall HSP60 protein in activated TM was found in mitochondria (Fig. 1D).

Our further step was to investigate whether increased HSP60 expression in CD4 and CD8 TM at 24 hours post-activation occurred in an HSF1-dependent manner. First, we assessed their intracellular expression for both total and active pS326 forms of HSF1. Although no difference was found for the expression of total HSF1 (Supplementary Fig. 1B), our data showed increased expression of HSF1 pS326 in activated TM when compared to non-activated cells [FI = 4.3 and 7.4, respectively for CD4 and CD8 T cells] (Fig. 1E)^33,34^. Interestingly, FI of HSF1 pS326 expression that were determined in CD4 and CD8 TM after 24 hours of T cell activation correlated with those of HSP60 (r = 0.74, *P* = 0.006) (Fig. 1F). As expected, we found lower expression of activated HSF-1 pS326 in CD45RA^+^ naïve T cells after TCR engagement, when compared to their TM counterparts [Fold decrease; FD = 4 and 2.4 respectively for CD4 and CD8 T cells] (Supplementary Fig. 1C). Then, we assessed whether inhibiting HSF1 activity in TM prevented their increased HSP60 expression during T cell activation. To inhibit HSF1 activity, we used two approaches: PBMC were T cell activated by anti-CD3/CD28 Abs in the presence of the selective HSF1 inhibitor KRIBB11 (KR)^42^; We also conducted specific *Hsf1* gene silencing on purified CD4 and CD8 TM before TCR engagement. At 24 hours post-activation, we determined that KR treatment led to approximately 77% inhibition of HSF1 pS326 expression in both CD4 and CD8 TM (Fig. 1G and Supplementary Fig. 1D). Additionally, specific *Hsf1* gene silencing led to more than 85% inhibition for both total and pS326 HSF1 proteins (Fig. 1G and Supplementary Fig. 1D). No difference was found between non electroporated [cells with TcR engagement alone] and Ctr siRNA-transfected TM. By using Annexin-V staining, we found that HSF1 inhibition in 24 h-long activated TM did not affect cell viability (Supplementary Fig. 1E). Our data further confirmed that the inhibition of HSF1 activity for 24 hours, especially by specific *Hsf1* gene silencing, prevented increased HSP60 expression in activated CD4 and CD8 TM, with intracellular levels that were comparable to non-activated cells (Fig. 1H).

**Fig. 2:**
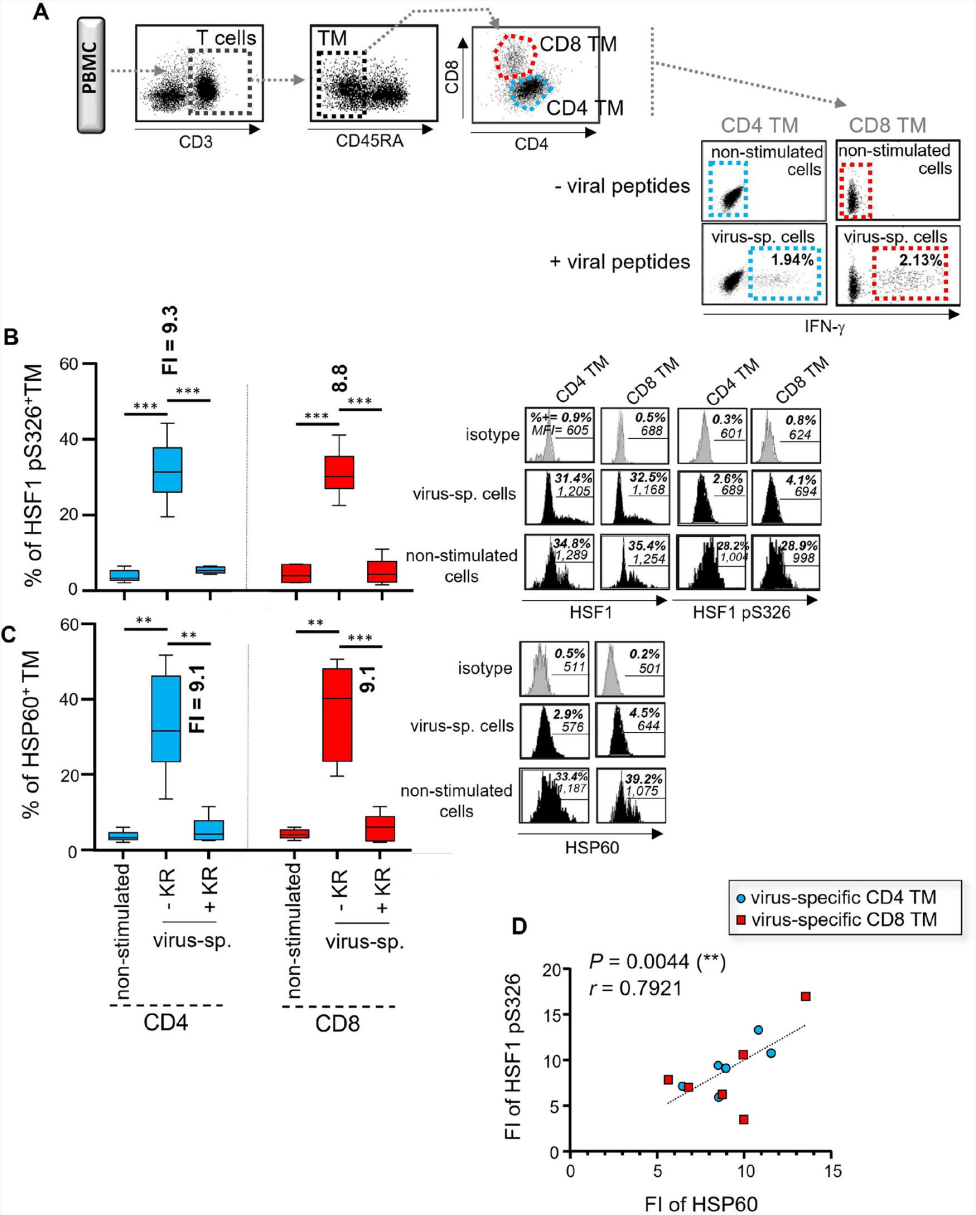
HSF1-dependent HSP60 increase is also confirmed in virus-specific CD4 and CD8 TM after peptide stimulation. Briefly, PBMC were antigen-specifically stimulated or not with viral peptide pools and anti-CD28 Abs for 24 hours, w/wo KR. **A** IFN-γ-based gating strategy to define virus-specific (virus-sp.) CD4 and CD8 TM after 24 hours of peptide stimulation, along with their non-stimulated cell counterparts. % of IFN-γ^+^ virus-sp. cells in CD4 and CD8 TM at 24 hours of peptide stimulation are also indicated in bold. **B** % of HSF1 pS326 [after normalization with HSF1 expression] and **C** HSP60 positivity in virus-sp. CD4 and CD8 TM w/wo KR, and in non-stimulated cells at 24 hours of culture (mean ± SD; *n* = 6); Fold increases (FI) of protein expression in CD4 and CD8 TM after 24 hours of viral peptide stimulation are also indicated in bold. FI of protein expression was calculated as follows: [% of protein positivity in virus-specific TM] / [% of protein positivity in non-stimulated TM]. Of note, representative black histograms of HSF1 (total and pS326 forms) and HSP60 are also shown for virus-sp. ± KR, and non-stimulated TM. **D** Correlation between FI of HSF1 pS326 and HSP60 expression after 24 hours of peptide stimulation. *N* = 12 virus-sp. TM.

Finally, we investigated whether HSF1-dependent increase of HSP60 expression was also found in virus-specific TM after peptide stimulation. At 24 hours post-peptide stimulation, we identified virus-specific CD4 and CD8 TM by their positive staining for IFN-γ (Fig. 2A)^13,14,36^. When compared to non-stimulated cells, virus-specific CD4 and CD8 TM displayed similar expression of total HSF1 (Supplementary Fig. 1F) but had a significant increase for both HSF1 pS326 and HSP60 (Fig. 2B and C). Like TM that were TCR engaged with anti-CD3/CD28 Abs, since we found a positive correlation between FI of HSF1 pS326 and HSP60 expression in virus-specific TM after peptide stimulation (r = 0.79, *P* = 0.004) (Fig. 2 D). With more than 82% inhibition of HSF1 pS326 expression (Fig. 2B), the addition of KR during the peptide stimulation prevented increased HSP60 expression in virus-specific CD4 and CD8 TM, resulting in low levels that were comparable to non-stimulated cells (Fig. 2 C). Once again, KR treatment of virus-specific TM did not influence their levels of cell apoptosis at 24-hour postculture (Supplementary Fig. 1G).

Altogether, our data show that, after TCR engagement, both CD4 and CD8 TM, including virus-specific cells, increase mitochondrial HSP60 expression in a time- and HSF1-dependent manner [with a maximum of protein expression at 24 hours post-T cell activation]. In addition, we confirm that adding KR for 24 hours led up to 93% inhibition of HSP60 protein expression in activated TM with no impact on cell viability.

### HSP60 controls the glutaminolysis-driven mitochondrial ATP generation in activated CD4 TM

**Fig. 3:**
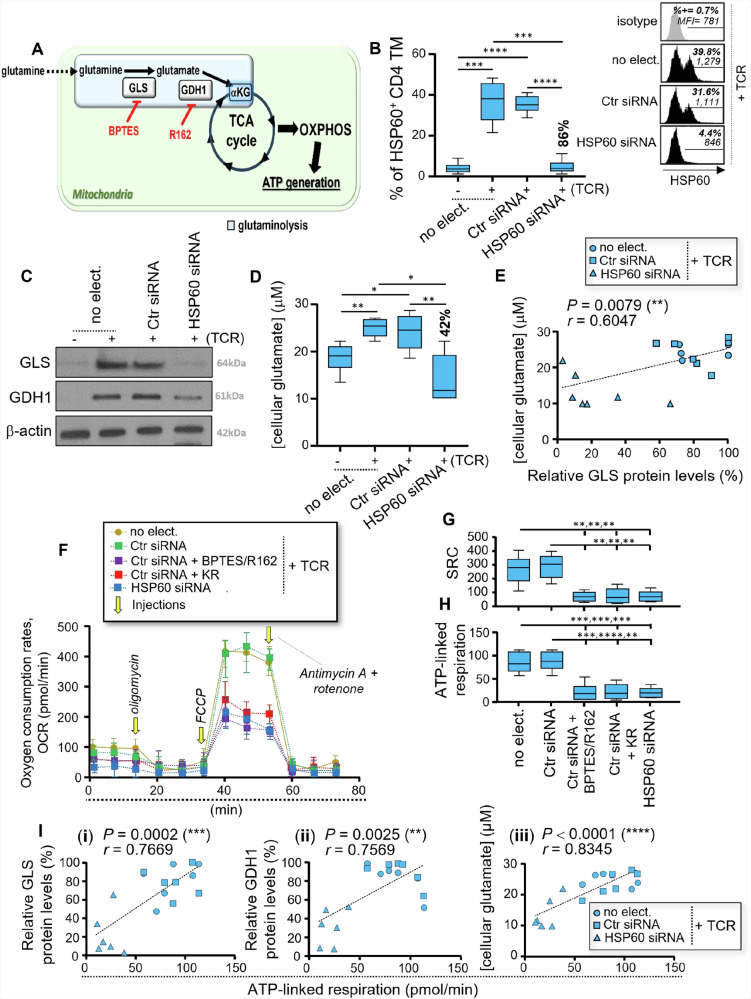
HSP60 controls the levels of mitochondrial ATP generation in activated CD4 TM through glutaminolysis. **A** Schematic of glutaminolysis [mitochondrial conversion of glutamine to αKG], which is supported by GLS and GDH1 enzymes and can be inhibited with BPTES and R162 drugs (indicated in red). **B–I** Briefly, purified CD4 TM were pre-transfected with Ctr siRNA or HSP60 siRNA or were not, after which they were cultivated w/wo anti-CD3 and CD28 Abs for 24 hours. **B** % of HSP60^+^ CD4 TM including no electroporation (no-elect.) control as well as cell transfection conditions with Ctr siRNA or HSP60 siRNA at 24 hours of culture. Inhibition % of HSP60 expression for specific *Hspd1* silencing is indicated in bold. Representative black histograms are also shown for activated CD4 TM w/wo specific *Hspd1* gene silencing including the grey isotype control. **C** Representative GLS and GDH1 blots in CD4 TM including β-actin at 24 hours of culture (from 6 independent experiments). **D** Assessment of cellular glutamate concentrations by Bioluminescence-based assay at 24 hours of culture [with R162 treatment]. Inhibition % of glutamate concentrations that are driven by specific *Hspd1* silencing is also indicated in bold. **E** Correlation between cellular glutamate concentrations and relative GLS protein levels at 24 hours post-activation. *N* = 18 CD4 TM. **F–I** Assessment of whole mitochondrial respiration in activated CD4 TM w/wo specific *Hspd1* gene silencing. KR treatment as well as glutaminolysis inhibition by BPTES/R162 were also conducted on Ctr siRNA-transfected cells. **F** Representative respiratory kinetics for each study condition. **G** Spare respiratory capacity (SRC) and **H** ATP-linked respiration determined in pmol/min. **I** Correlation between levels of mitochondrial ATP-linked respiration and **(i)** GLS or **(ii)** GDH1 protein levels, or **(iii)** cellular glutamate concentrations at 24 hours post-activation. *N* = 18 CD4 TM w/wo specific *Hspd1* gene silencing. **B**, **D**, **G**, **H** Results shown are the mean relative ± SD of *n* = 6.

CD4 TM, including virus-specific cells, preferentially use glutaminolysis to fuel mitochondrial ATP generation^13^. Glutaminolysis is a two-step mitochondrial enzymatic process, where glutamine is first converted to glutamate by the enzyme glutaminase (GLS) and then to αKG by glutamate dehydrogenase 1 (GDH1) (Fig. 3A). Recently, mitochondrial GLS and GDH1 enzymes have both been identified as HSP60 interactors^27^.

Therefore, we first assessed the impact of specific *Hspd1* gene silencing in CD4 TM on GLS and GDH1 protein levels at 24 hours post-activation. By using flow cytometry, we first confirmed that specific *Hspd1* gene silencing led to around 86% inhibition of HSP60 expression in activated CD4 TM (Fig. 3B), with no impact on their cell apoptosis levels (Supplementary Fig. 2A). Of note, no difference for HSP60 expression was found between non-electroporated and control small interfering RNA (Ctr siRNA)-transfected CD4 TM at 24 hours post-activation (Fig. 3B). Our western blot analysis indicated that TCR engagement of CD4 TM drove both GLS and GDH1 protein levels to increase (Fig. 3C and Supplementary Fig. 2B). Specific *Hspd1* gene silencing [HSP60 inhibition] led to more than 66% inhibition for both GLS and GDH1 proteins in activated CD4 TM (Fig. 3C and Supplementary Fig. 2B). Of note, HSP60 inhibition in activated CD4 TM did not impact the surface levels of the glutamine/amino-acid transporter ASCT2 (Supplementary Fig. 2B). To further assess the impact of specific *Hspd1* gene silencing on glutaminolysis, CD4 TM were T cell activated or not with anti-CD3/CD28 Abs in the presence of the selective GDH1 inhibitor R162. Indeed, by preventing the enzymatic transformation of glutamate with R162 (Fig. 3A), we could appreciate its cellular levels in CD4 TM at 24 hours of culture. First, we confirmed that activated CD4 TM display higher levels of cellular glutamate when compared to non-activated cells (Fig. 3D). Our data further show that specific *Hspd1* gene silencing in activated CD4 TM led to approximately 42% inhibition of glutamate production. As expected, glutamate levels in activated CD4 TM with/without (w/wo) specific *Hspd1* gene silencing [HSP60 inhibition] correlated with their GLS protein levels (r = 0.60, *P* = 0.008) (Fig. 3E). We then investigated whether specific *Hspd1* gene silencing impacted autophagy in CD4 TM, since autophagy has a great influence on glutaminolysis-dependent ATP generation by providing cells with free glutamine^13^. For this, we used two complementary autophagy assessments, both of which were the most indicative of its induction in CD4 and CD8 TM after TCR engagement^13,14^. First, we evaluated the percentages (%) of autophagic TM by flow cytometry, by characterizing them with autophagy-related gene 1 (ATG1) and Beclin-1 dual-staining. We further determined their lytic autophagy activity in culture, as previously described^13,14^. Our analysis showed that, although we confirmed increased autophagy in activated CD4 TM when compared to non-activated cells^13^, specific *Hspd1* gene silencing had no influence on autophagy at 24 hours post-activation (Supplementary Figs. 2C and D). Together, our results reveal that HSP60 is a key regulator of the protein levels of glutaminolytic enzymes within CD4 TM after TCR engagement, as well as glutamate production, without influencing autophagy.

Next, we evaluated whether HSP60 regulated whole mitochondrial respiration and ATP generation in activated CD4 TM by using a Seahorse XF metabolic flux analyzer. We also included Ctr siRNA-transfected CD4 TM that were activated for 24 hours with KR. Following the manufacturer’s instruction, the respiratory kinetics that resulted from the sequential addition of pharmacological agents that target components of the electron transport to the cells allowed us to calculate both the mitochondrial spare respiratory capacity (SRC) and ATP-linked respiration (Supplementary Fig. 2E). Aside specific *Hspd1* gene silencing and KR treatment, glutaminolysis was also inhibited in activated CD4 TM with BPTES/R162 co-treatment and used as positive controls for reduced SRC and ATP-linked respiration (Fig. 3A)^13^. Indeed, our previous data always confirmed potent inhibition of mitochondrial energy production in CD4 TM when their glutaminolysis pathway was blocked by using BPTES/R162 co-treatment^13^. First, we confirmed similar respiratory profiles between non-electroporated and Ctr siRNA-transfected CD4 TM at 24 hours post-activation (Fig. 3F-H). As expected, there was a potent reduction of mitochondrial respiration [SRC and ATP-linked respiration] in activated CD4 TM when the glutaminolysis pathway was inhibited in cell culture (Fig. 3F-H). Our data further show that specific *Hspd1* gene silencing and KR treatment [HSP60 inhibitory drug] both led to reduced SRC and mitochondrial ATP-linked respiration, resulting in low levels that were comparable to glutaminolysis inhibition (Fig. 3F-H). Levels of mitochondrial ATP-linked respiration in activated CD4 TM w/wo HSP60 silencing correlated with both GLS and GDH1 protein levels (r = 0.77 and 0.76, *P* = 0.0002 and 0.003) and cellular glutamate concentrations (r = 0.83, *P* < 0.0001) (Fig. 3I).

In summary, HSP60, whose increased protein expression in CD4 TM after TCR engagement can be prevented by specific *Hspd1* gene silencing and KR treatment, is a critical metabolic regulator of mitochondrial ATP generation through glutaminolysis.

### HSP60 is also required for proper FAO-mediated ATP generation in CD8 TM after TCR engagement

**Fig. 4:**
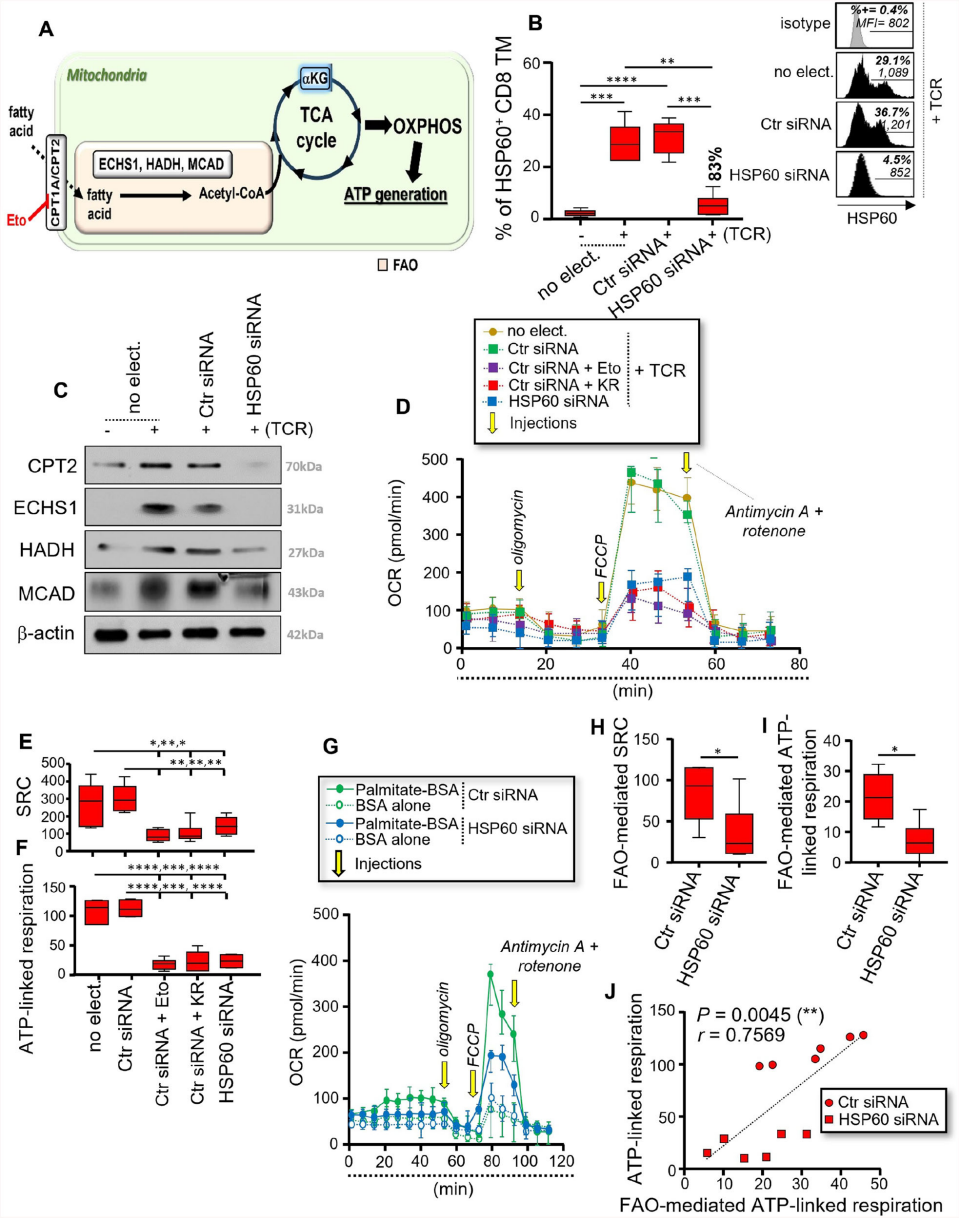
HSP60 is a key regulator of the FAO-driven mitochondrial ATP generation in activated CD8 TM. **A** Schematic of FAO [mitochondrial conversion of fatty acids], which is supported by several metabolic enzymes. This includes the CPT1A inhibitor etomoxir (Eto) in red. **B**–**J** Purified CD8 TM were pre-transfected with Ctr siRNA or HSP60 siRNA or were not, after which they were cultivated w/wo anti-CD3 and CD28 Abs for 24 hours. **B** % of HSP60^+^ CD8 TM including no electroporation (no-elect.) control as well as cell transfection conditions with Ctr siRNA or HSP60 siRNA at 24 hours of culture. Inhibition % of HSP60 expression for specific *Hspd1* silencing is indicated in bold. Representative black histograms are also shown for activated CD8 TM w/wo specific *Hspd1* gene silencing including the grey isotype control. **C** Representative CPT2, ECHS1, HADH, and MCAD blots in CD8 TM including β-actin at 24 hours of culture (from 6 independent experiments). **D**–**F** Assessment of whole mitochondrial respiration in activated CD8 TM w/wo specific *Hspd1* gene silencing, KR and Eto. **D** Representative respiratory kinetics for each study condition. **E** SRC and **F** ATP-linked respiration determined in pmol/min. **G**–**I** Assessment of FAO-mediated mitochondrial respiration, by using Seahorse XF Palmitate Oxidation Stress test kit and long-chain palmitate substrate. **G** Representative respiratory kinetics of activated CD8 TM w/wo specific *Hspd1* gene silencing, and when the nutrient restriction step was conducted either with palmitate-BSA substrate or BSA alone. **H** FAO-mediated SRC and **I** FAO-mediated ATP-linked respiration in pmol/min. Of note, FAO-mediated values were calculated as follows: [value with palmitate-BSA] – [value with BSA alone]. **J** Correlation between the whole and FAO-mediated ATP-linked respiration at 24 hours post-activation. *N* = 12 CD8 TM w/wo specific *Hspd1* gene silencing. **B**, **E**, **F**, **H**, **I** Results shown are the mean relative ± SD of *n* = 6.

Our previous work has shown that activated CD8 TM, including virus-specific cells, favors FAO to generate mitochondrial ATP^14^. FAO is the major pathway for fatty acid degradation. Briefly, fatty acids enter mitochondria via the lipid transporters (CPT1A/CPT2) where they are then oxidized through FAO into two carbon Acetyl-CoA molecules, which can enter the TCA cycle to generate ATP (Fig. 4A). FAO involves multiple enzymes, including ECHS1, HADH and MCAD, which have all been listed as HSP60 interactors along with CPT2^27^.

Therefore, we first subjected purified CD8 TM to specific *Hspd1* gene silencing to investigate its impact on the protein levels of several FAO enzymes [ECHS1, HADH and MCAD], and CPT2 lipid transporter. Like CD4 TM, specific *Hspd1* gene silencing in CD8 TM led to a potent 83% inhibition of intracellular HSP60 expression after TCR engagement without influencing the levels of cell apoptosis (Fig. 4B and Supplementary Fig. 2F). Our western blot analysis revealed increased protein levels for CPT2 and all FAO enzymes in CD8 TM after 24 hours of T-cell activation when compared to non-activated cells (Fig. 4C and Supplementary Fig. 2G). Specific *Hspd1* gene silencing led to 55-72% inhibition for the FAO-related proteins (Fig. 4C and Supplementary Fig. 2G). As with CD4, we found that TCR engagement of CD8 TM drove increases for both the % of autophagic cells and lytic autophagy activity^14^, which were not influenced by specific *Hspd1* gene silencing (Supplementary Figs. 2H and I). Together, our results showed that HSP60 regulates the proteins levels of several FAO-related enzymes and CPT2 lipid transporter but does not influence autophagy after TCR engagement.

Next, we assessed the whole mitochondrial respiration and ATP generation in activated CD8 TM w/wo specific *Hspd1* gene silencing and KR. Ctr siRNA-transfected CD8 TM that were T cell activated in the presence of Eto, a selective FAO inhibitor which is widely used for its irreversible inhibitory effects on CPT1A, were included here as a positive control for reduced SRC and ATP-linked respiration (Fig. 4A)^14^. First, we confirmed the same SRC and ATP-linked respiration between non-electroporated and Ctr siRNA-transfected CD8 TM at 24 hours post-activation (Fig. 4D-F). We further found that specific *Hspd1* gene silencing and KR treatment in CD8 TM led to reduced SRC and ATP-linked respiration, resulting in low levels that were comparable with Eto inhibiting the FAO (Fig. 4D-F). Finally, the Agilent Seahorse XF Palmitate Oxidation Stress Test kit, which provides validated reagents to measure long-chain FAO via assessment of changes in oxygen consumption (OCR) in intact cells, was used here in activated CD8 TM w/wo specific *Hspd1* gene silencing. The protocol relies on the cell capacity to oxidize the fatty acid palmitate in culture when other exogenous substrates are limited. In this context, 45 minutes before mitochondrial respiratory assessment, CD8 TM were incubated with bovine serum albumin (BSA)-palmitate saturated fatty acid complex or BSA alone in a substrate-limited media. Our data show that HSP60 inhibition in CD8 TM led to potent reductions for both the FAO-dependent SRC and ATP-linked respiration (Fig. 4G-I). Interestingly, the levels of FAO-mediated ATP-linked respiration in CD8 TM w/wo specific *Hspd1* gene silencing correlated with those of ATP-linked respiration that were determined by assessing whole mitochondrial respiration (r = 0.76, *P* = 0.005) (Fig. 4J). This supported the critical role of the FAO and its regulation by HSP60 in fueling the overall mitochondrial ATP generation in CD8 TM after T cell activation^14,43,44^.

Overall, our data confirm a key role of HSP60 in supporting the FAO-driven mitochondrial ATP generation in CD8 TM after TCR engagement, which can be counteracted by specific *Hspd1* gene silencing and KR treatment.

### HSP60 is requested for mitochondrial ATP-dependent T cell effector functions like IL-21 and cytotoxic molecule production in CD4 and CD8 TM respectively

We and others have shown that, although Ag-specific CD4 and CD8 TM initiate an early metabolic reprogramming after TCR engagement that is characterized by aerobic glycolysis to produce IFN-γ, TNF-α and IL-2 cytokines^5–7,10,45^; they further rely on the use of mitochondrial ATP generation for other specific effector functions such as IL-21, Perforin and Granzyme-B expressions^11,13,14,16^.

**Fig. 5:**
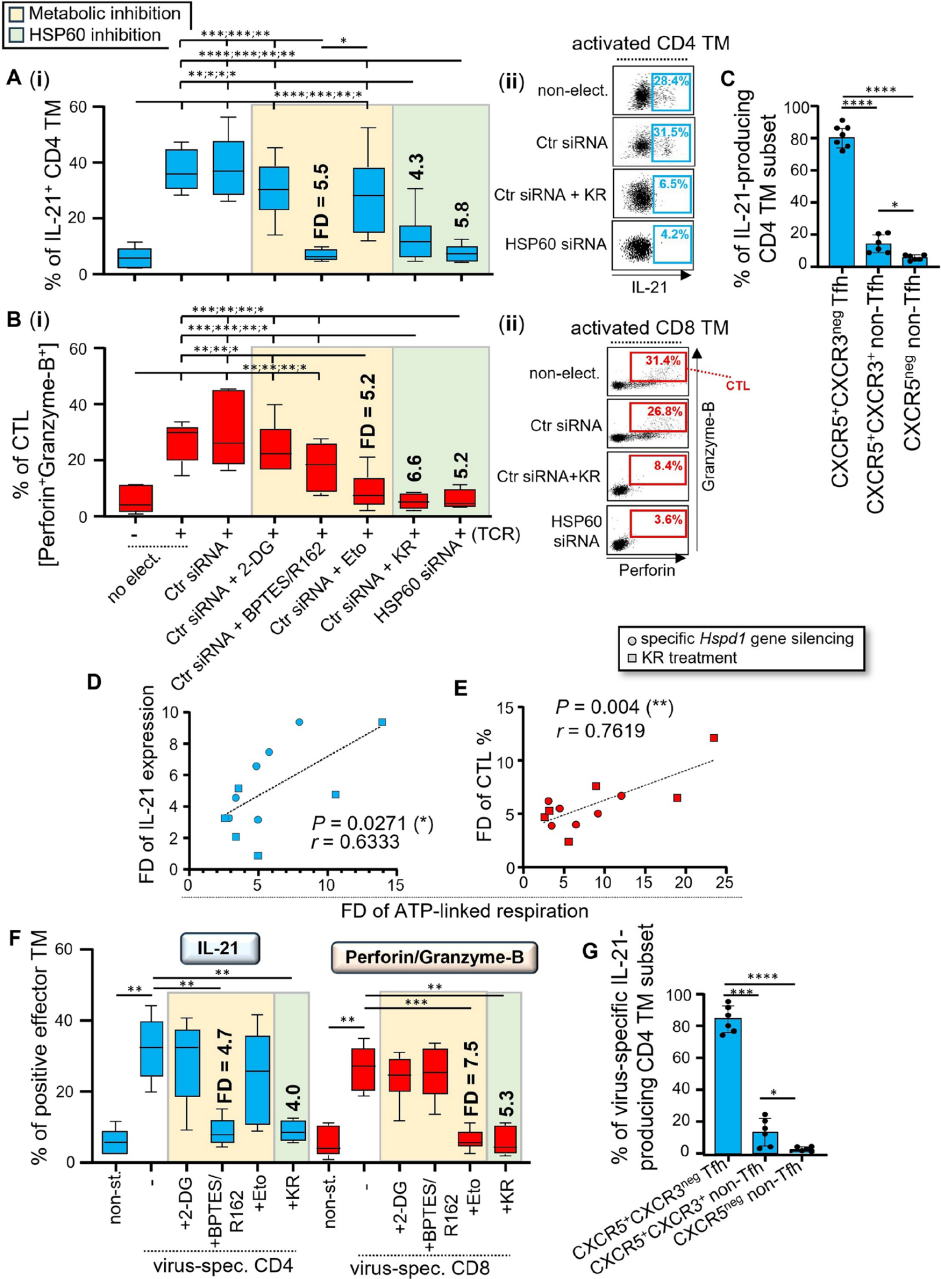
HSP60 regulates mitochondrial ATP-dependent IL-21 cytokine and cytotoxic molecule production in virus-specific TM. **A**, **B** Purified CD4 and CD8 TM were pre-transfected with Ctr siRNA or HSP60 siRNA or were not, after which they were cultivated w/wo anti-CD3/CD28 Abs for 24 hours, w/wo KR, 2-DG, BPTES/R162 and Eto. **A** % of IL-21^+^ CD4, and **B** Perforin^+^Granzyme-B^+^ CTL TM at 24 hours of culture. Results shown are **(i)** the mean ± SD of n = 6, and **(ii)** representative dot plots of IL-21 expression in activated CD4, and of Perforin/Granzyme-B dual-staining in CD8 TM, when cells were cultivated w/wo specific *Hspd1* gene silencing and KR. Fold decreases (FD) of T cell effector functions in the presence of glutaminolysis, FAO and HSP60 inhibition are also indicated in bold. **C** Proportion of CXCR5^+^CXCR3^neg^ Tfh and non-Tfh cells [CXCR5^neg^ and CXCR5^+^CXCR3^+^ cells] whitin IL-21-producing CD4 TM at 24 hours post-activation. **D**, **E** Correlation between FD of mitochondrial ATP-linked respiration and those of the % of **D** IL-21^+^ CD4 TM, or **E** CTL TM at 24 hours post-activation. *N* = 12 TM per figure. **F**, **G** Briefly, PBMC were stimulated or not with viral peptides for 24 hours, w/wo KR and metabolic inhibitors [2-DG, BPTES/R162 and Eto]. **F** At 24 hours of culture, virus-specific cells were assessed for their % of IL-21^+^ virus-specific CD4 and CTL TM. FD of virus-specific effector functions are also indicated in bold. **G** Proportion of Tfh and non-Tfh cells in IL-21-producing virus-specific CD4 TM at 24 hours post-peptide stimulation. **A–C**, **F**, **G** Results are the mean ± SD of *n* = 6.

Therefore, we assessed whether reduced mitochondrial ATP generation in activated TM, which was driven by specific *Hspd1* gene silencing and KR treatment (Figs. 3 and 4), influenced IL-21 production in CD4 and the dual-expression of cytotoxic Perforin and Granzyme-B molecules in CD8 TM [cell subset defined as CTL]. Briefly, purified CD4 and CD8 TM were pre-transfected with Ctr siRNA or HSP60 siRNA or were not, after which they were cultivated w/wo anti-CD3 and CD28 Abs for 24 hours w/wo KR. We also included Ctr siRNA-transfected TM that were activated for 24 hours with different metabolic inhibitors including 2-DG [glycolysis], BPTES/R162 [glutaminolysis], and Eto [FAO inhibition]. As expected, our flow cytometry analysis confirmed increased expression for all effector molecules (IL-21, Perforin, Granzyme-B, IFN-γ, TNF-α, and IL-2) in activated TM at 24 hours post-culture when compared to non-activated cells (Fig. 5A and B; and Supplementary Figs. 3A-C). Of note, the levels of T cell effector functions in non-electroporated TM were comparable to those of Ctr siRNA-transfected cells at 24 hours post-activation. Our data further confirm that, after TCR engagement, both CD4 and CD8 T-cells expressed IFN-γ, TNF-α and IL-2 exclusively in a glycolysis-dependent manner (Supplementary Figs. 3A-C). Conversely, inhibiting glutaminolysis or FAO pathways during T cell activation, but not glycolysis, respectively, led to defective IL-21 production in CD4 TM and, Perforin and Granzyme-B dual-expression in CTL; the low levels of which were comparable to non-activated cells (Fig. 5A and B)^13,14^. In fact, our results show that both specific *Hspd1* gene silencing and KR treatment in activated TM led to more than 4.3- and 5.2-fold decrease (FD) of their % of IL-21 producing CD4 and CTL cells (Fig. 5A and B), without affecting the glycolytic expression of IFN-γ, TNF-α and IL-2 cytokines (Supplementary Figs. 3A-C). No difference was found for the low levels of IL-21, Perforin and Granzyme-B expressions between TM that have been T cell activated in the presence of HSP60 inhibition and those of non-activated cells. Of note, approximately 80% of IL-21-producing CD4 TM at 24 hours post-activation were CXCR5^+^CXCR3^neg^ T follicular helper (Tfh) cells (Fig. 5C), same subset that was defined by the highest levels of HSP60 induction after TCR engagement (Fig. 1 C). Interestingly, our data show that FD of IL-21 and Perforin/Granzyme-B expressions correlated to those of mitochondrial ATP-linked respiration in both HSP60-depleted CD4 and CD8 TM (r = 0.63 and 0.76, *P* = 0.027 and 0.004) (Fig. 5D and E).

Finally, we investigated whether HSP60 inhibition in virus-specific CD4 and CD8 TM, when they were stimulated 24 hours with viral peptides and KR, also led to defective cell ability to produce IL-21, Perforin and Granzyme-B. Once again, we stimulated virus-specific CD4 and CD8 TM in the presence of 2-DG, BPTES/R162, and Eto. Similarly to what we found with TM that were TCR-engaged, IL-21 production in virus-specific CD4 TM occurred in a glutaminolysis-dependent manner, whereas virus-specific CTL immunity was only impaired with FAO inhibition by Eto (Fig. 5F)^13,14^. We found that KR treatment of virus-specific TM led to approximately 4- and 5.3-FD of the % of IL-21-producing CD4 T cells and CTL, comparable to non-stimulated cells (Fig. 5F). Same as for CD4 TM that were TCR-engaged, the majority of IL-21-producing cells in virus-specific CD4 TM was CXCR5^+^CXCR3^neg^ Tfh cells (84.1%; Fig. 5G). Of note, HSP60 inhibition in virus-specific TM did not impact their glycolytic induction of IFN-γ, TNF-α and IL-2 cytokines (Supplementary Figs. 3, D-F).

Altogether, our data show that activated TM including virus-specific cells express IFN-γ, TNF-α and IL-2 in a glycolysis-dependent manner, whose cytokine levels are not affected despite HSP60 inhibition. However, the glutaminolytic IL-21 production which is mainly found in Tfh cells, and the FAO-dependent dual-expression of Perforin/Granzyme-B in CTL are both prevented during TCR engagement by specific *Hspd1* gene silencing and KR treatment.

### HSP60 inhibition leads to reduced αKG levels in both activated CD4 and CD8 TM which can be supplemented with DMKG

αKG is a rate-determining intermediate in the mitochondrial tricarboxylic acid (TCA) cycle and has a crucial role in the cellular energy metabolism, including ATP generation^46,47^.

**Fig. 6:**
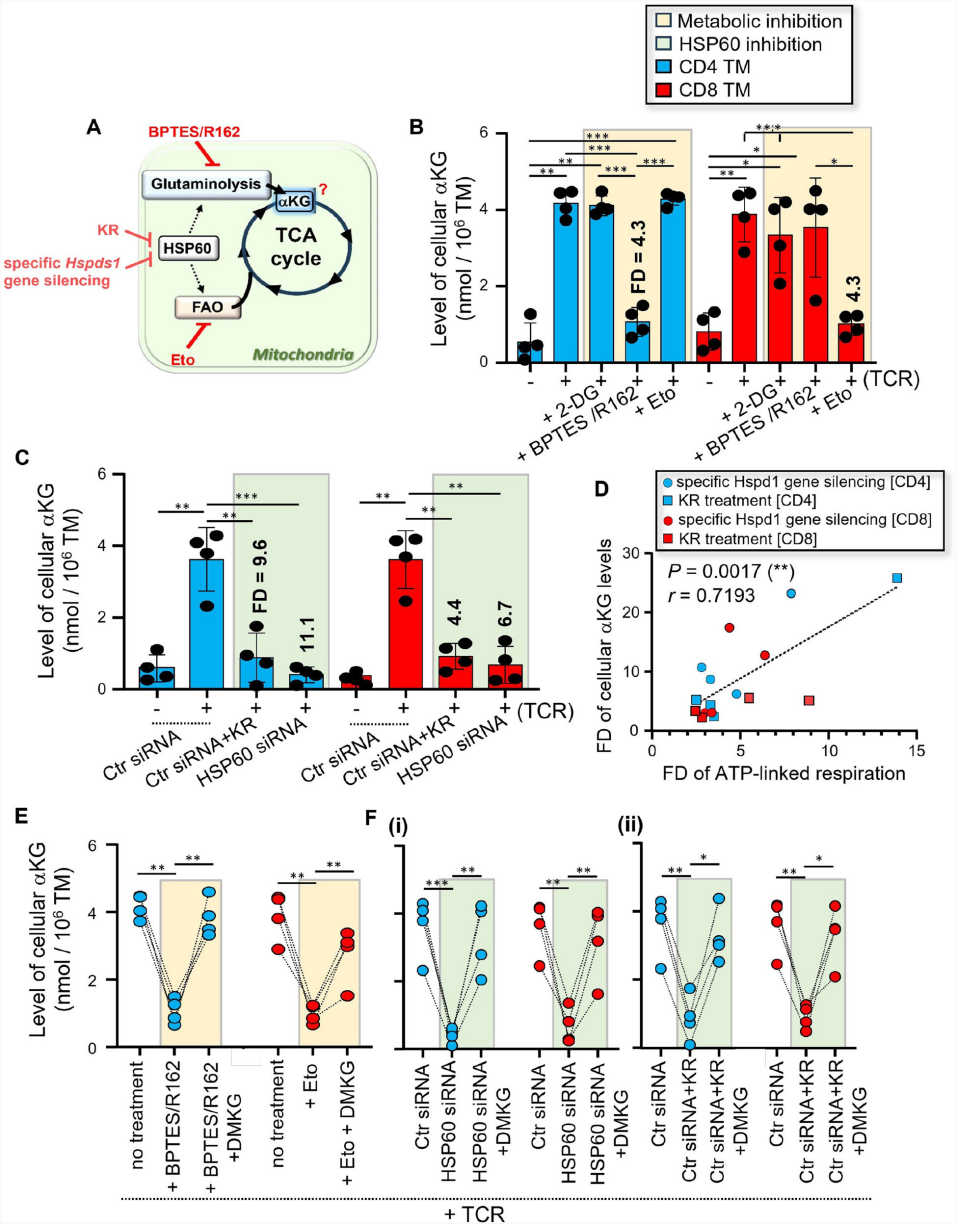
HSP60 inhibition in both CD4 and CD8 TM leads to reduced endogenous αKG levels after TCR engagement. **A** Assessment in activated CD4 and CD8 TM of their cellular levels of TCA intermediate αKG in the context of glycolysis, glutaminolysis, FAO and HSP60 inhibition. **B** Purified CD4 and CD8 TM were activated or not with anti-CD3/CD28 Abs for 24 hours w/wo metabolic inhibitors [2-DG, BPTES/R162, Eto and Rot/AMA]. Then, we assessed the cellular αKG levels both in CD4 and CD8 TM for each study condition. Fold decreases (FD) of cellular αKG levels, when CD4 and CD8 TM were activated with BPTES/R162 and Eto, respectively, are also indicated in bold. **C** Purified TM were pre-transfected with Ctr siRNA or HSP60 siRNA, after which they were activated or not with anti-CD3/CD28 Abs for 24 hours w/wo KR. Results represent the cellular levels of αKG in non-activated and activated CD4 and CD8 TM w/wo HSP60 inhibition, and also include the FD of αKG levels when TM were activated in the presence of specific *Hspd1* gene silencing and KR. **D** Correlation between FD of αKG levels and ATP-linked respiration at 24 hours post-activation. *N* = 16 activated TM w/wo specific *Hspd1* gene silencing and KR. **E** Cellular αKG levels in BPTES/R162-treated CD4 and Eto-treated CD8 TM, when cells were T cell activated for 24 hours w/wo DMKG. **F** Cellular αKG levels in HSP60-depleted CD4 and CD8 TM, when cells were T cell activated for 24 hours w/wo DMKG; **(i)** Specific *Hspd1* gene silencing, and **(ii)** KR treatment. **A–C**, **E**, **F** Data shown are the mean ± SD of *n* = 4.

Taking this into account, we assessed whether glycolysis [2-DG], glutaminolysis [BPTES/R162], FAO [Eto] and HSP60 inhibition [specific *Hspd1* gene silencing and KR] influenced the levels of cellular αKG in activated TM (Fig. 6A). Briefly, purified CD4 and CD8 TM were pre-transfected with Ctr siRNA or HSP60 siRNA or were not, after which they were cultivated w/wo anti-CD3/CD28 Abs for 24 hours w/wo KR and metabolic inhibitors [2-DG, BPTES/R162, and Eto]. First, we found that 24 hours of T cell activation led to increased levels of cellular αKG in both CD4 and CD8 TM when compared to non-activated cells (Fig. 6B and C). Of note, similar αKG levels were found between non-electroporated and Ctr siRNA-transfected TM at 24 hours post-activation. Our data showed that increased αKG levels, which were found in activated CD4 and CD8 TM, were prevented when cells were TCR-engaged respectively with BPTES/R162 and Eto, but not with 2-DG, thereby displaying low levels like non-activated cells (Fig. 6B). Our analysis further revealed that increased αKG levels in CD4 and CD8 TM during T cell activation were also prevented by HSP60 inhibition [Fold decreases (FD) = 11.1 and 6.7, and 9.6 and 4.4, respectively for specific *Hspd1* gene silencing and KR in activated CD4 and CD8 TM] (Fig. 6C). In fact, we found that FD of endogenous αKG levels in activated TM, when cells were T cell activated without HSP60, correlated to those of mitochondrial ATP-linked respiration (r = 0.72, *P* = 0.002) (Fig. 6D).

Next, we assessed whether supplementing cultures with DMKG, which is a cell-permeable diester analog of αKG^32^, improved the reduced levels of endogenous αKG found in activated TM when they were T cell activated in the presence of glutaminolysis [CD4], FAO [CD8] and HSP60 inhibition. Not only our data confirm that DMKG was effective in replenishing the reduced levels of cellular αKG, which were found in BPTES/R162-treated CD4 and FAO-treated CD8 TM after TCR engagement (Fig. 6E), but also rescued those mediated by HSP60 inhibition (Fig. 6F).

Altogether, our results show that cellular levels of endogenous αKG, which were increased in both CD4 and CD8 TM after 24 hours of TCR engagement when compared to non-activated cells, were fully prevented with HSP60 inhibition but could be replenished by supplementing T cells with DMKG.

### DMKG supplementation rescues mitochondrial ATP-dependent IL-21 and cytotoxic molecule production in both virus-specific CD4 and CD8 TM

Recent studies have shown that supplementing CD4 TN with DMKG results in higher levels of mitochondrial ATP-linked respiration after TCR engagement, despite glutamine restriction in cell culture^48,49^. Therefore, in the last section of experimentation, we decided to assess whether DMKG supports mitochondrial ATP-dependent IL-21 production in Tfh cells and, Perforin and Granzyme-B dual-expression in CTL as well.

**Fig. 7:**
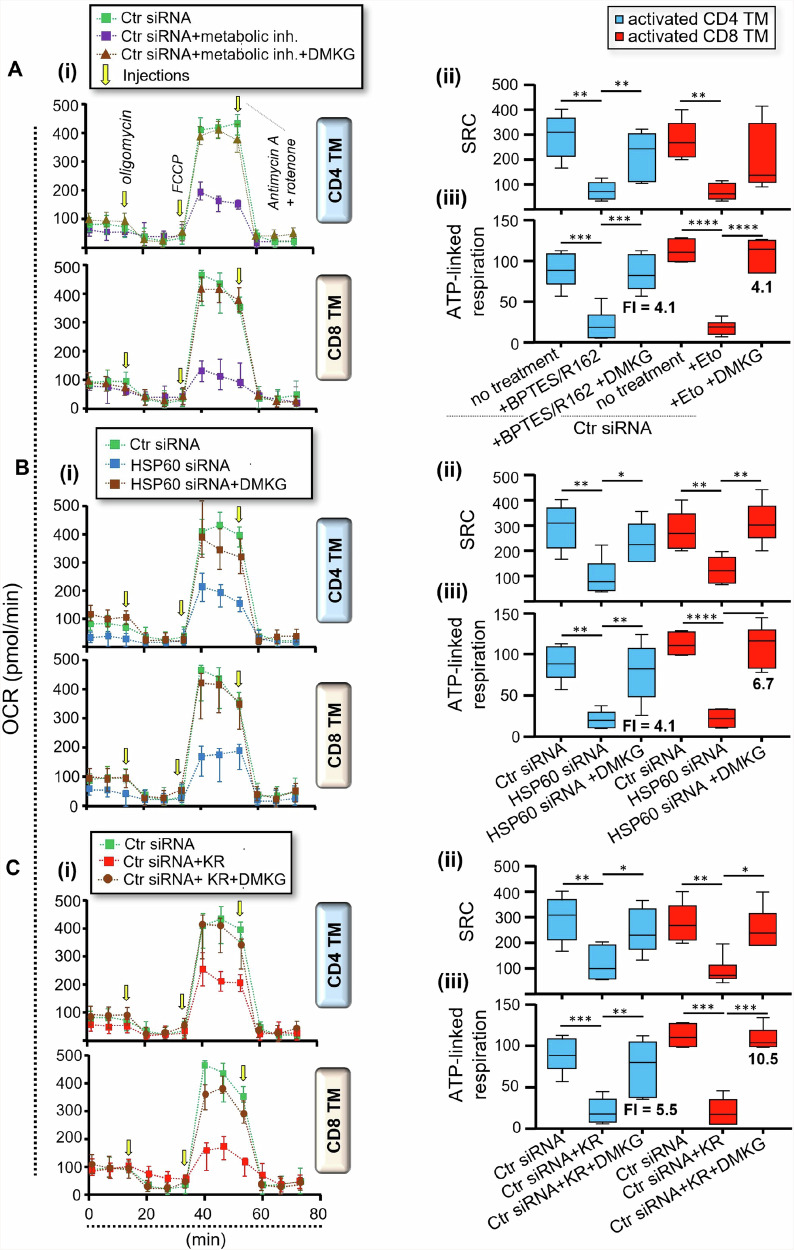
DMKG rescues the reduced levels of mitochondrial ATP generation in TM when activated in the presence of HSP60 inhibition. Briefly, purified CD4 and CD8 TM were pre-transfected with Ctr siRNA or HSP60 siRNA, after which they were T cell activated with anti-CD3/CD28 Abs for 24 hours w/wo BPTES/R162 [CD4], Eto [CD8], KR and DMKG. At 24 hours post-activation, CD4 and CD8 TM were collected to assess the mitochondrial SRC and ATP-linked respirations. Data shown are the metabolic assessments that were conducted on **A** Ctr siRNA-transfected TM w/wo metabolic inhibitors (metabolic inh.) and DMKG, **B** TM w/wo specific *Hspd1* gene silencing and DMKG, and **C** Ctr siRNA-transfected TM w/wo KR and DMKG. **A**–**C (i)** Representative respiratory kinetics for each study condition, **(ii)** SRC and (**iii)** ATP-linked respiration (pmol/min). Fold increases (FI) of mitochondrial ATP-linked respiration in activated TM with DMKG supplementation are also indicated in bold. Results shown for **(ii, iii)** are expressed as the mean ± SD of *n* = 6.

First, purified CD4 and CD8 TM were pre-transfected with Ctr siRNA or HSP60 siRNA or were not, after which they were T cell activated with anti-CD3/CD28 Abs for 24 hours w/wo BPTES/R162 [CD4], Eto [CD8], KR and DMKG. At 24 hours post-activation, cells were collected to evaluate whole mitochondrial respiration (SRC and ATP-linked respiration) in the presence or absence of DMKG supplementation. Our data show that DMKG was effective in rescuing the levels of mitochondrial ATP-linked respiration when CD4 and CD8 TM were T-cell activated in the presence of glutaminolysis and FAO inhibition, respectively (Fig. 7A). Similarly, we found that DMKG led to a full rescue of the mitochondrial ATP-linked respiration in activated CD4 and CD8 TM, not only in the context of specific *Hspd1* gene silencing [fold increases (FI) = 4.1 and 6.7] (Fig. 7B), but also when cells underwent KR treatment [FI = 5.5 and 10.5] (Fig. 7C). FI of ATP-linked respiration in activated TM when HSP60-depleted cells were supplemented with DMKG correlated to those of endogenous αKG levels at 24 hours of culture (r = 0.63, *P* = 0.0081) (Supplementary Fig. 4A). Of note, DMKG supplementation in HSP60-depleted CD4 and CD8 TM neither influenced their reduced HSP60 expression (Supplementary Fig. 4B) nor levels of cell apoptosis at 24 hours post-activation (Supplementary Fig. 4C). Altogether, our results show that DMKG supplementation can counteract the reduction of both endogenous αKG levels and related mitochondrial ATP generation in activated CD4 and CD8 TM despite HSP60 inhibition.

**Fig. 8:**
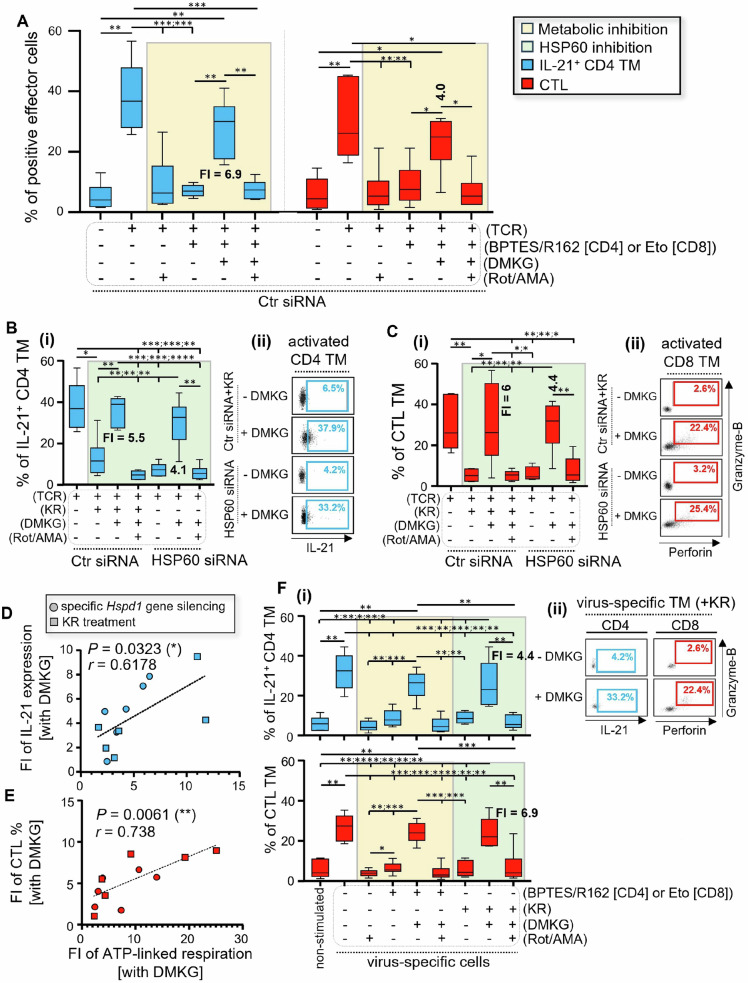
DMKG rescues ATP-dependent IL-21 and cytotoxic molecule production in virus-specific TM despite glutaminolysis, FAO and HSP60 inhibition. **A–E** Purified CD4 and CD8 TM were pre-transfected with Ctr siRNA or HSP60 siRNA, after which they were T cell activated or not with anti-CD3/CD28 Abs w/wo BPTES/R162 [CD4], Eto [CD8], KR, DMKG and Rot/AMA. **A** % of IL-21-producing CD4 TM and **B** CTL in Ctr siRNA-transfected TM were determined at 24 hours of cell culture w/wo metabolic inhibitors. Fold increases (FI) of T cell effector functions when DMKG was supplementing cell cultures are also indicated in bold. FI were calculated as follows: [% of effector TM with DMKG] / [% of effector TM without DMKG]. **(C)** IL-21 production in CD4 TM and, **D** Perforin and Granzyme-B co-expression in CTL, when 24 h-long of TCR engagement were conducted w/wo specific *Hspd1* gene silencing, KR, DMKG and Rot/AMA. Results shown are **(i)**% of positive effector T cells, and **(ii)** representative dot plots in HSP60-depleted CD4 and CD8 TM w/wo DMKG supplementation. FI of these effector T cell functions in HSP60-depleted CD4 and CD8 TM with DMKG supplementation are also indicated in bold. **D**, **E** Correlations between FI of ATP-linked respiration and those of **D** IL-21 production in CD4, or **E** Perforin/Granzyme-B dual-staining in CD8 TM with DMKG supplementation. *N* = 12 activated TM w/wo specific *Hspd1* gene silencing and KR at 24 hours of culture. **F** PBMC were stimulated or not with viral peptides for 24 hours w/wo glutaminolysis or FAO inhibition, and w/wo KR, DMKG and Rot/AMA. **(i)**% of IL-21^+^ CD4 and CTL T cells in non-stimulated and virus-specific TM at 24 hours of culture. FI of T cell effector functions in virus-specific CD4 and CD8 TM with DMKG supplementation are also indicated in bold. **(ii)** representative dot plots for virus-specific IL-21^+^ CD4 and CTL TM, when cells were virally stimulated with KR and w/wo DMKG. **A–C**, **F**, Data shown are expressed as the mean ± SD of *n* = 6.

Next, Ctr siRNA- and HSP60-transfected CD4 and CD8 TM were T cell activated or not with anti-CD3/CD28 Abs for 24 hours w/wo BPTES/R162 [CD4], Eto [CD8], KR and DMKG, but also w/wo rotenone (Rot) and antimycin A (AMA) co-treatment to inhibit OXPHOS-driven mitochondrial ATP generation. At 24 hours of culture, cells were collected to assess their intracellular levels of IL-21 expression in CD4 and, Perforin and Granzyme-B dual-expression in CD8 TM. First, our data confirm that IL-21 and cytotoxic molecule production, which was increased in activated CD4 and CD8 TM, respectively, were prevented with glutaminolysis [CD4], FAO [CD8], OXPHOS and HSP60 inhibition (Fig. 8A-C). We found that DMKG supplementation rescued both IL-21-producing CD4 and CTL TM, when activated in the presence of BPTES/R162 and Eto respectively (Fig. 8A). DMKG was also effective in restoring these effector functions in activated TM despite HSP60 inhibition (Fig. 8B and C). In fact, our analysis at 24 hours post-activation revealed that DMKG supplementation in HSP60-depleted TM led to approximately a 4.1- and 4.4-fold increase (FI) of IL-21 production in CD4 and a 5.5- and 6-FI of Perforin/Granzyme-B co-expression in CD8 T cells respectively with specific *Hspd1* gene silencing and KR treatment (Fig. 8B and C). FI of mitochondrial ATP-linked respiration in HSP60-depleted TM that were activated with DMKG correlated with those of IL-21 expression in CD4 (r = 0.62, *P* = 0.03) and, Perforin and Granzyme-B co-staining in CTL T cells (r = 0.74, *P* = 0.006) (Fig. 8D and E); This indicated that DMKG-driven rescue of these T cell effector functions likely occurred in an ATP-dependent manner. In this context, we confirm that DMKG-driven rescue of IL-21 and cytotoxic molecule production in activated CD4 and CD8 TM were, as expected, abrogated when cell culture was conducted under OXPHOS inhibition by Rot/AMA (Fig. 8A-C). Of note, DMKG and Rot/AMA neither influenced HSP60 expression, nor levels of cell apoptosis in both activated CD4 and CD8 TM at 24 hours of culture (Supplementary Fig. 4B and C).

Finally, we investigated whether DMKG supplementation further enhanced the intracellular production of IL-21 cytokine and cytotoxic molecules of virus-specific CD4 and CD8 TM in an ATP-dependent manner. To do so, PBMC were stimulated or not with viral peptides for 24 hours w/wo KR, DMKG and Rot/AMA. At 24 hours of culture, our flow cytometry analyses reveal that, same as for TM that were T cell activated with anti-CD3/CD28 Abs, the expression levels of IL-21, Perforin and Granzyme-B in virus-specific CD4 and CD8 TM were reduced in the presence of glutaminolysis [CD4], FAO [CD8] and HSP60 inhibition, resulting in low levels that were similar to non-stimulated cells (Fig. 8F). However, our data confirm that DMKG was effective in rescuing IL-21 and cytotoxic molecule production in virus-specific TM despite glutaminolysis, FAO and HSP60 inhibition by KR (FI = 4.4 and 6.9, respectively for the % of IL-21^+^ CD4 and CTL T cells). Similarly to TCR-engaged TM, our analysis shows that DMKG-driven rescue of virus-specific T cell effector functions occurred in an ATP-dependent manner, since they disappeared in the presence of OXPHOS inhibition (Fig. 8F). We found similar levels of cell apoptosis in virus-specific CD4 and CD8 TM w/wo DMKG and Rot/AMA at 24 hours of culture (Supplementary Fig. 4D). Of note, more than 82.7% of IL-21-producing virus-specific CD4 TM, when activated in the presence of HSP60 inhibition but rescued by DMKG, were Tfh cells (Supplementary Fig. 4E).

Overall, the last section of results shows that impaired IL-21 and cytotoxic molecule production in CD4 and CD8 TM after TCR engagement, including virus-specific cells, which were mediated by glutaminolysis [CD4], FAO [CD8] and HSP60 inhibition, could be rescued by DMKG in a mitochondrial ATP-dependent manner (Supplementary Fig. 5).

## Discussion

Today, it is well acknowledged that cellular metabolism, which includes the generation of ATP molecules dictates T cell fate after TCR engagement, such as cell growth, survival and effector immune functions, which are all energy-dependent biological processes^3,4,43–45,50–53^. There are two primary and overlapping metabolic pathways in eukaryotic cells for generating ATP: glycolysis and mitochondrial OXPHOS. Evidence shows that, in a few minutes after TCR engagement, Ag-specific CD4 and CD8 TM perform aerobic glycolysis^8^, which allows them to rapidly keep up with ATP demands whilst preserving the biosynthetic nature of mitochondria to generate new material for cell growth^10,54–56^. Aerobic glycolysis in early T cell activated CD4 and CD8 TM is requested to produce pro-inflammatory IFN-γ, TNF-α, and IL-2 cytokines^5–7,10^ (Supplementary Fig. 3). In the meantime, starting at 6 hours and until days 3 post-activation, IFN-γ-producing TM including virus-specific cells further use mitochondrial OXPHOS to support other T cell effector functions^7,11–15^. These functions include the expression of IL-21 cytokine, mainly produced in peripheral CXCR5^+^CXCR3^neg^CD4 T follicular (Tfh) cells [> 80% of IL-21-producing CD4 TM], and the dual-expression of Perforin and Granzyme-B molecules in CD8 cytotoxic T lymphocytes (CTL), both occurring in a mitochondrial ATP-dependent manner^13,14^. Virus-specific TM do not need glycolysis for mitochondrial ATP generation, but rather use additional nutrients than glucose in the form of glutamine and fatty acids, which are both provided by autophagy after TCR engagement^13,14,16,21,57^. Precisely, with IL-21-producing Tfh cells, including virus-specific cells, glutamine enters the mitochondria where it is first converted into glutamate by GLS^13^ and then into the rate-determining TCA cycle intermediate αKG by GDH1 that ultimately fuels mitochondrial ATP generation. At 24 hours post-TCR engagement, not only does our data confirm that CD4 TM display increased cellular levels for both glutamate and αKG metabolites, but also higher protein levels for GLS and GDH1 enzymes, as well as glutaminolysis-dependent ATP-linked respiration when compared to non-activated cells^13^. In CTL, fatty acids enter the mitochondria through the CPT1A and CPT2 lipid transporters where they are then transformed into Acetyl-CoA molecules through FAO, which involves several enzymes such as ECHS1, HADH and MCAD. In this context, our data confirmed that activated CD8 TM have higher expression levels for CPT2, ECHS1, HADH and MCAD proteins as well as FAO-driven ATP-linked respiration when compared to non-activated cells. Although not intuitive at first, since Acetyl-CoA fuels the TCA cycle two steps prior to that of the αKG intermediate^58^, we found nonetheless that the increased cellular levels of αKG, which are found in CD8 TM at 24 hours post-activation, are fully prevented when activated in the presence of FAO inhibition by Eto, which yields low αKG levels comparable to those of non-activated cells.

Herein, we identify HSP60 chaperone as a key regulator of mitochondrial ATP generation in TM after TCR engagement, by bolstering glutaminolysis and FAO pathways in virus-specific Tfh cells and CTL, respectively. HSP60, which is a molecular chaperone mainly located in the mitochondria, interacts, and assists in the folding of a multitude of proteins in the mitochondrial matrix^25–27^. The latter include CPT2 lipid transporter and multiple enzymes of the glutaminolysis and FAO pathways, some of which have even been confirmed in cancer cells with specific *Hspd1* gene silencing^28,30,59,60^. Our data showed that the mitochondrial expression of HSP60 in both virus-specific CD4 and CD8 TM is induced after TCR engagement in an HSF1-dependent manner^33^ and achieves the highest level of protein expression at 24 hours post-activation. Through specific *Hspd1* gene silencing and cell treatment with the selective HSF1 inhibitor KR, which led up to 96.5% and 94% of HSP60 inhibition in activated CD4 and CD8 TM respectively, we found that the chaperone is a key regulator of their mitochondrial energy metabolism. Similarly to cancer cells^28–30^, HSP60 inhibition by specific *Hspd1* gene silencing in TM leads to a potent inhibition of the protein levels for GLS and GDH1 in activated CD4 as well as CPT2, ECHS1, HADH and MCAD enzymes in CD8 T cells, thus resulting in low levels of these proteins that were comparable to non-activated cells. In fact, we found that HSP60 inhibition in activated CD4 TM not only results in decreased glutamate production, but also leads to lower glutamate/glutamine ratio (Supplementary Fig. 6), which indicated a contributive role of HSP60 chaperone on glutamine consumption during TCR engagement. However, to quantitatively and precisely determine how HSP60 inhibition impacts the engagement of glutaminolysis [CD4] and FAO [CD8] metabolic pathway during TCR engagement, it may be informative to use isotope-tracing metabolomics on cultivated cells (glutamine or fatty acids), and further assess their transformations by mass spectrometry.

HSP60 inhibition in cancer cells further leads to reduced cellular αKG levels and mitochondrial ATP generation^31,59–62^. As expected, our data confirmed that HSP60 inhibition in activated CD4 and CD8 TM, including virus-specific cells, leads to reduced levels of cellular glutamate and TCA cycle αKG as well as mitochondrial ATP-dependent IL-21 and cytotoxic molecule production. It is worth noting that, in effector CD4 and CD8 TM, both HSP60 inhibition and glutaminolysis [CD4]/FAO [CD8] blockade during TCR engagement resulted in potent inhibition of αKG levels even though this TCA-related metabolite can be the by-product of many different metabolic pathways. Of note, HSP60 inhibition in activated CD4 and CD8 TM did not impact the glycolysis-dependent production of IFN-γ, TNF-α, and IL-2 cytokine. Since HSP60 can interact with hundreds of mitochondrial interactors in cancer cells^27^, we cannot exclude that, aside regulating mitochondrial ATP generation in CD4 and CD8 TM after TCR engagement, HSP60 could also influence their survival and cell growth in culture. To verify if it is the case, we assessed the levels of cell apoptosis and cell numbers per 10^6^ PBMC in both CD4 and CD8 TM after T cell activation at 24-, 48- and 72-hours w/wo specific *Hspd1* gene silencing. First, we confirmed increased numbers for both CD4 and CD8 TM at 72 hours post-activation, which confirms T cell growth after TCR engagement at longer time periods of culture (Supplementary Fig. 7). However, HSP60 inhibition in activated TM neither influenced the levels of cell apoptosis nor cell numbers per million PBMC up to 72 hours post-activation (Supplementary Fig. 6). In summary, HSP60 regulates mitochondrial ATP generation in IL-21-producing Tfh cells and CTL, including virus-specific cells, by assisting the glutaminolysis- and FAO-dependent fueling of endogenous αKG.

DMKG, the diester and cell-permeable analog of αKG, has recently been shown to significantly contribute carbon to mitochondrial TCA cycle metabolism in multiple cancer cells by supplementing cellular levels of αKG^32^. Interestingly, previous data confirm that DMKG supplementation further rescued reduced αKG levels and mitochondrial ATP-linked respiration in CD4 TN, when activated under glutamine restriction^48,49^. In the current study, DMKG supplementation was further found to replenish reduced αKG levels in virus-specific TM, when activated for 24 hours in the presence of glutaminolysis [CD4], FAO [CD8] and HSP60 inhibition. As a result, we confirm that DMKG supplementation represents an effective way to rescue mitochondrial ATP-dependent IL-21 and cytotoxic molecule production in virus-specific Tfh cells and CTL respectively. Of note, DMKG is still able to fully restore mitochondrial ATP-dependent T cell effector functions in CD4 and CD8 TM, when activated in HSP60-inhibited conditions up to 72 hours of culture (Supplementary Fig. 5). This indicates that HSP60 inhibition in activated TM at 24 to 72 hours of culture has no impact on the final steps of ATP generation that involve the OXPHOS respiratory complexes, contrary to what has been found in cancer cells^29,31,63,64^. Aside DMKG, acetate supplementation could also be another way to rescue mitochondrial ATP generation in virus-specific CTL cells when necessary. Indeed, by fueling the cellular levels of Acetyl-CoA, acetate supplementation of tumor-specific CD8 T cells metabolically bolsters their T cell effector immunity^65^. Acetate dietary supplementation in mice also improves Granzyme-B expression in influenza virus-specific CD8 T cells by enhancing the cellular levels of TCA cycle αKG and FAO-driven mitochondrial ATP-linked respiration^66^. In summary, we show that DMKG supplementation is an enticing therapeutic concept to boost antiviral T cell immunity in a mitochondrial ATP-dependent manner, if they had defective glutaminolysis or FAO pathways w/wo impaired HSP60 expression.

Since they play a key protective role in viral infections, including human immunodeficiency virus type 1 (HIV-1), gaining information on how IL-21-producing Tfh cells and CTL metabolically control their virus-specific immunity will provide insights towards the development of new improved T cell-based therapies by identifying specific molecular targets^67,68^. Our previous works, by using the same in vitro model of T cell responses to viral peptides, have shown that inducing the catabolic process of autophagy with drugs is an enticing therapeutic venue to consider for boosting both HIV-1-specific IL-21 and cytotoxic molecule production in people living with HIV-1 (PLWH)^13,14,16,57^. Defective HIV-1-specific Tfh and CTL responses, which are still found in PLWH despite long-term intake of suppressive antiretroviral therapy (ART)^11,13,14,18–20,69^, can be improved by autophagy inducer AICAR treatment, resulting in better down-stream mitochondrial process of ATP generation in the same fashion than EC^13,14^. Importantly, our data were confirmed to be accurate since stimulating autophagy with drugs is confirmed effective in improving both in vivo CD4 and CD8 T cell responses against HIV-1 in ART-treated HIV^+^ humanized mice, and even delays viral rebound after ART cessation^70^. Here, we propose DMKG supplementation as a new mean to restore optimal ATP-dependent Tfh and CTL immunity against viruses when cells cannot properly fuel mitochondrial activity through the TCA cycle for ATP production. In this context, on a total of 12 ART-treated individuals, we have selected two of them, whose defective HIV-1-specific IL-21 and cytotoxic molecule production was not rescued by AICAR treatment. Not only our preliminary data confirm impaired HSP60 expression in both HIV-1-specific Tfh cells and CTL for these two individuals, but also validate that DMKG supplementation rescues their defective anti-HIV-1 T cell responses when AICAR treatment fails to do so (Supplementary Fig. 8). This indicate that DMKG supplementation might be a better way to restore effective HIV-specific Tfh and CTL immunity in PLWH than autophagy inducing drugs, because it overcomes not only autophagy defects, but also impaired HSP60 expression when found in PLWH after T cell activation. Furthermore, our data must be considered for optimizing T-cell immunity to viral vaccines, particularly in the context of poor-responder vaccines to seasonal influenza viruses. Peripheral CXCR5^+^CXCR3^neg^ Tfh cells, which represent more than 80% of IL-21-producing CD4 TM in our cell culture, positively influence B-cell Ab production and CTL activity during viral infections^14,39^. In fact, IL-21 production by Tfh cells and related Granzyme-B by CTL are the most highly indicative features of vaccine efficacy against influenza viruses, especially in PLWH^71–74^. Since DMKG supplementation improves Tfh- and CTL-related immunity in virus-specific T cells, including those against influenza virus, it may be considered as a vaccine adjuvant to seasonal Flu.

To conclude, we provide clear evidence that virus-specific CD4 and CD8 TM must properly generate mitochondrial ATP through glutaminolysis and FAO pathways, both of which depend on HSP60 induction for optimal enzyme expression after TCR engagement, and whose ATP-dependent Tfh cell- and CTL-related immunity can fully be rescued by DMKG supplementation (Supplementary Fig. 5).

## Materials and methods

### Products, drug inhibitors and reagents

RPMI-1640 media, FBS, antibiotics and PBS were obtained from Wisent Inc. The selective HSF1 inhibitor KRIBB11 (KR), FAO inhibitor etomoxir (Eto), glutaminolysis inhibitors BPTES and R162, glycolysis inhibitor 2-deoxy-D-glucose (2-DG), OXPHOS-related complex I inhibitor rotenone (Rot), OXPHOS-related complex III inhibitor antimycin A (AMA), autophagy-related lysosome inhibitor Bafilomycin A1 (BaF), DMKG, and IL-21 cytokine, were all provided from Sigma-Aldrich. KR, Eto, BPTES, R162, 2-DG, Rot, AMA, BaF, DMKG, and IL-21 were used in our study at: [2 μg/mL, 5 μM, 30 μM, 30 μM, 0.5 mM, 5 μM, 1 μM, 10 ng/mL, 20 μg/mL, and 10 ng/mL].

### Memory T cell (TM) purification

Leukapheresis from healthy subjects (middle-aged men [25 to 50 years old]) were all provided by J.P. Routy and A. Massicote, McGill University Health Centre, Montreal, Quebec, Canada. Each donor signed informed consent forms approved by the McGill University Health Centre research ethics board [ethic number: 2021-7111]. First, PBMC were isolated by a Ficoll-Hypaque (GE Healthcare) density gradient. CD4 or CD8 TM were then purified from PBMC using the untouched EasySep^TM^ Human Memory CD4 + T Cell and Memory CD8 + T Cell Enrichment Kits (StemCell Technologies), respectively. For isolation of mitochondrial fractions, we chose to purify the overall TM population (CD4 and CD8 included): we performed an initial negative selection using Human Pan T Cell Isolation kit (Miltenyi Biotec), which allowed us to retain total T cells, followed by another negative selection using CD45RA MicroBeads (Miltenyi Biotec), which allowed us to purify the overall CD45RA^neg^TM. Our protocols allowed for more than 94.6% purification with any cell stimulation and apoptosis^13,14,35,36^.

### T-cell activation methods

CD4 and CD8 TM were activated in complete RPMI [10% FBS; penicillin/streptomycin], by using two complementary methods. In the first, for transcribing the events of TCR engagement, we activated PBMC or purified TM [CD4, CD8, or total] with 1 μg⧸mL anti-CD3 and 2 μg⧸mL anti-CD28 Abs (BD Biosciences). In the second, to study virus-specific TM, PBMC were stimulated with two pools of viral peptides; Specifically, cells were activated with 2 μg⧸mL of CEFTA:CD4 [35 MHC Class II-restricted peptides from human cytomegalovirus (CMV), Epstein-Barr virus (EBV), influenza viruses, and adenovirus 5; Mabtech] and CEF:CD8 extended [32 MHC Class I-restricted peptides from human CMV, EBV and influenza virus; Mabtech] peptide pools, with 2 μg⧸mL anti-CD28 Abs. These viral peptide pools are considered to be the gold standard to test the functionality of Ag-specific T cells and, as such, are typically included as a positive controls in most antiviral T cell assays^14,75,76^. Both virus-specific CD4 and CD8 TM were determined in culture by positive staining for IFN-γ, as previously described^13,14,36^. Importantly, our experimental design has proven that we can trigger the same molecular and metabolic mechanisms that contribute to mitochondrial ATP generation in CD4 and CD8 T cells between Ag-specific cells via the stimulation with viral peptides and the whole TM after TCR engagement with anti-CD3/CD28 Abs^13,14,36^. Finally, cultures of purified CD8 TM were stimulated with IL-21 as previously described^14^. This was mandatory to ensure proper autophagy-driven glutamine and fatty-acid production after TCR engagement.

### Specific *Hsf1* and *Hspd1* [HSP60 inhibition] gene silencing

First, we purified a minimum of 10^7^ CD4 or CD8 TM, and electroporated them using Nucleofector II technology according to the Amaxa Biosystems manufactor’s protocol. Specific siRNAs for HSF1 (siRNA IDs: 115674 [siRNA 1] and 3139 [siRNA 2]), for HSP60 (IDs: 11067 [siRNA 1] and 11163 [siRNA 2]), and negative control siRNA (ID: 4390843) were all obtained from ThermoFisher Scientific. Of note, 5 μg of siRNA were transfected or not for 2 h without antibiotics. Cells were washed three times thereafter, to remove dead necrotic cells, counted and incubated for 24 h. Finally, TM were T-cell activated and collected to assess metabolic and immune features. The efficacy of HSF1 and HSP60 protein inhibition under specific gene silencing was systematically confirmed by flow cytometry.

### Multiparameter flow cytometry

Supplementary Table 1 shows the multiparameter antibody (Ab) cocktails that we developed in our study. Of note, GolgiPlug (BD Biosciences, 555029) and GolgiStop (BD Biosciences, 554724) were always added in culture 6 hours before assessing cytokines and cytotoxic molecules. **Intracellular staining (ICS):** To assess the expression levels of autophagy-related genes (ATG1 and Beclin-1), HSF1, HSP60, and effector molecules (IL-21, Perforin, Granzyme-B as well as IFN-γ, IL-2 and TNF-α), we subjected cells to ICS as previously described^13,14,35,36^. Briefly, after surface staining with specific antibodies for TM phenotyping, we fixed and then permeabilized the cells with 0.25% saponin (Sigma Aldrich, 47036) before the intracellular staining per se. After three washes, stained cells were finally ready for flow cytometry analyses. The viability marker 7-aminoactinomycin D or 7-AAD (ThermoFisher Scientific, 00-6993-50) was used to exclude dead cells from analysis. **PhosFlow protocol:** PhosFlow was performed to assess the intracellular expressions of HSF1 pS326 (the phosphorylated and active form of HSF1) in TM. Briefly, cellular fixation was done by using 4% PFA for 10 min at 36 °C followed by surface staining for 10 min at 4 °C. Afterwards, the cellular permeabilization was done using 90% ice-cold methanol for 30 min at 4 °C followed by 30 min of intracellular staining in PBS + 2% FBS at room temperature. Once again, 7-AAD was used again to exclude dead cells from analyses. **Apoptosis assessment:** As previously described^35,36^, the percentages of apoptotic cells were determined in gated CD4 and CD8 TM with Annexin-V-V450 labeling (BD Biosciences), and by using Annexin V Binding Buffer (BD Biosciences). **Data analyses:** BD LSRII Fortessa flow cytometer (BD) was used to collect the data which was analyzed using the DIVA software. 200,000-500,000 gated cells were analyzed for each sample.

### Western blotting

Purified CD4 or CD8 TM were activated or not w/wo specific *Hspd1* gene silencing. TM were then collected and subjected to SDS-PAGE for Western blot analysis, as previously explained in other publications^13,38,77–79^. Briefly, we collected 10 µg of protein lysates per culture condition to determine the levels of glutaminolysis-related enzymes [GDH1 and GLS], FAO-related enzymes [ECHS1, HADH, and MCAD] and lipid transporter CPT2, respectively in CD4 and CD8 TM. All primary Abs including anti-β-actin Abs (monoclonal rabbit Ags), and secondary HRP-conjugated goat anti-mouse and anti-rabbit IgG Abs were all purchased from ThermoFisher Scientific. To maximize detection sensitivity, we used the Clarity Max ECL substrates from BioRad. Of note, densitometric quantifications for all proteins of interest (normalized to β-actin whose expression level was used as loading control) were determined using Image J software.

### Mitochondria isolation

10^8^ purified TM were activated with anti-CD3/CD28 Abs for 24 hours and then collected to gather purified fractions of mitochondria using the Mitochondria Isolation kit for Cultured Cells (ThermoFisher Scientific; Cat number: 89874). To achieve high purity for both the mitochondrial and cytosolic fractions from cell lysates, we followed the manusfacturer’s instruction with the traditional Dounce homogenization-based protocol. Finally, western blot analyses of purified mitochondria and cytosolic fractions were conducted to determine protein levels of HSP60, mitochondrial lipid transporter CPT2 and cytosolic peroximosal membrane protein 70 (PMP70). CPT2 and PMP70 allowed us to respectively confirm high purity of the collected mitochondrial and cytosolic fractions. All primary monoclonal rabbit and secondary HRP-conjugated Abs were purchased from ThermoFisher Scientific.

### Assessment of cellular levels of glutamate and glutamine [CD4 TM]

Purified CD4 TM were activated or not for 24 hours w/wo specific *Hspd1* gene silencing. Of note, the GDH1 inhibitor R162 was added at the beginning in the cell culture to prevent the enzymatic conversion of cellular glutamate to αKG. Cells were then collected at 24 hours of culture to assess their intracellular, and accumulated, concentrations of glutamate and glutamine by using the Bioluminescence-based assay Glutamine/Glutamate-Glo assay kit (Promega; Cat number: J8021)^13^. We also determined all ratio [glutamate]/[glutamine] to evaluate the glutamine consumption in culture.

### Assessment of cellular levels of αKG [CD4 and CD8 TM]

Purified CD4 and CD8 TM were activated or not in the presence of OXPHOS inhibition by Rot/AMA co-treatment^7^. Of note, TM were activated w/wo specific *Hspd1* gene silencing, KR, 2-DG, BPTES/R162 [CD4] and Eto [CD8]. At 24 hours of culture, TM were washed three times in PBS to be subjected to colorimetric measurement of their intracellular αKG levels using the Alpha Ketoglutarate Assay kit (Abcam). Briefly, 2.10^6^ cells per culture condition were collected, homogenized, sonicated, and centrifugated in ice cold AKG assay buffer to get clear protein samples. The latter were thereafter subjected to perchloric acid-mediated deproteinization protocol to remove most of their proteins and stabilize the small molecules including cellular metabolites. The deproteinized samples and αKG standard [0 to 10 nM] were all resuspended in 50 µL αKG assay buffer in 96 well-microplate and incubated 45 minutes in a 37 °C CO_2_ incubator with 50 µL Colorimetric Reaction Mix. This step allowed the enzymatic conversion of αKG into pyruvate, which then transforms the colorless OxiRed probe to a color one (optic density; OD detectable at 570 nM). Of note, since TM are expected to have endogenous pyruvate that may generate unwanted background, we also incubated 50 µL of each deproteinized samples with 50 µL of Background Mix. The latter will assess in each samples the OxiRed probe transformation in the absence of αKG conversion. Finally, we determined all OD_570nM_ for AKG standard and the deproteinized samples, including those incubated with the Background mixes by using a Multiskan FC microplate reader (ThermoFisher). By referring to the standard curve, we finally determined the quantities of cellular αKG in nmol per million TM with the following formula: [αKG] = [(OD_570nM_ of sample incubated in Colorimetric Mix) – (OD_570nM_ of sample in Background Mix)] / 2.

### Metabolic flux assay (whole and FAO-mediated mitochondrial respirations)

At 24 hours post-activation, both CD4 and CD8 TM were collected to assess both whole and FAO-driven mitochondrial respiration, by using a Seahorse XF_96_ metabolic analyzer. Of note, 4.10^5^ cells per condition were needed to ensure reproducible observations [in triplicate]. **Whole mitochondrial respiration:** As previously explained^13,14^, TM were seeded on XF_96_ plates (Agilent Technologies) in complemented Agilent RPMI [Glucose 10 mM, Glutamine 2 mM and Pyruvate 1 mM]. The XF Cell Mito Stress Test kit was used according to the manufacturer protocol. Oxygen consumption rate (OCR) was determined under basal conditions and in response to respiration modulators that were injected during the assay to observe key parameters of mitochondrial energy metabolism. The modulators included in this assay were oligomycin, carbonyl cyanide 4-(trifluoromethoxy) phenylhydrazone (FCCP), Rotenone and Antimycin A (1.5 µM, 2 µM and 0.5 µM, respectively). We determined the spare respiratory capacity (SRC) and ATP-linked respiration for each condition respectively as follows: [maximal OCR determined after FCCP treatment]—[basal OCR determined before oligomycin treatment] and [basal OCR]—[minimal OCR determined after Rotenone/Antimycin A co-treatment]. **FAO-mediated mitochondrial respiration:** To determine the FAO status in CD8 TM, we used the Seahorse XF Palmitate Oxidation Stress Test kit protocol with a minor modification^14^. Once again, 4.10^5^ cells were seeded on XF_96_ plates without going through the suggested nutrient restriction pre-step. This was made to keep a satisfying cell viability (superior to 90% of cell viability confirmed by flow cytometry). However, cells were still cultured under nutrient restriction, but at the end of culture and for 45 minutes. In this context, cells were cultured in a CO_2_-free incubator at 37°C with a pre-warmed XF FAO media containing only a necessary minimal content of glucose (2.5 mM) and 0.5 mM of L-carnitine (Agilent Technologies). L-carnitine is an amino-acid derivative that transports fatty acids into cells to be processed for energy. The nutrient restriction step was also conducted in the presence of lipid substrate palmitate-bovine serum albumin (BSA) complex or BSA alone. Once again, OCR values, which were determined under basal conditions and in response to respiration modulators, were used to calculate SRC and ATP-linked respiration for each culture condition. FAO-mediated parameters (SRC and ATP-linked respiration) were determined according to the manufacturer’s instructions and by the formula: [values with palmitate] – [values with BSA]. **Data normalization:** All values were normalized to the number of viable cell events per seeded well thanks to the CytoFLEX benchtop flow cytometer (Beckman Coulter).

### Lytic autophagy activity

Lytic autophagy-dependent degradation of long-lived proteins in TM was quantified as previously described^13,14^. The method is based on a pulse-chase approach, whereby cellular proteins are radiolabeled by [^14 ^C] valine (PerkinElmer, NEC291EU050UC). First, 1.10^6^ purified TM were seeded, and incubated for 18 h in complete RPMI with 0.2µCi/ml of L-[^14^C] valine to label intracellular proteins (Pulse media). Cells were then washed three times with PBS to eliminate any unincorporated radioactivity. The short-lived proteins were allowed to be degraded for 24 h in fresh RPMI (Chase media). After that, TM were activated for 24 hours, and then seeded in 10% of trichloroacetic acid (TCA) containing RPMI. After centrifugation, precipitated cells were washed twice with cold 10% TCA RPMI and dissolved in 0.2 M NaOH for 2 h. Of note, the supernatants contained the acid-soluble radioactivity fraction. Radioactivity was finally quantified by liquid scintillation counting. The rate of autophagy-dependent degradation of long-lived proteins was calculated from the ratio of the acid-soluble radioactivity in the medium to the one in the acid-precipitable cell fraction.

### Statistics and reproductivity

The experiments described herein rely on the comparison of TM metabolic and effector features between two different study conditions [w/wo specific gene silencing, drug inhibitors and DMKG supplementation]. In this context, we systematically used the two-sided Student paired *t* test to compare two different in vitro conditions. Assuming that we would detect more than 10% data difference between two culture conditions, a sample size of 6 individuals per study group have been calculated using the G*power software to ensure a statistical power superior to 90%. Spearman’s correlation test was used to identify the association between two variables. *P* values of less than 0.05 were considered as significant difference between two parameters. Of note, *, 0.05 > *P* > 0.01; **, 0.01 > *P* > 0.001; ***, 0.001 > *P* > 0.0001; and ****, *P* < 0.0001.

### Reporting summary

Further information on research design is available in the Nature Portfolio Reporting Summary linked to this article.

## Supplementary information

Transparent Peer Review file

Supplementary Material

Description of Additional Supplementary Materials

Supplementary Data

Reporting Summary

## Data Availability

All relevant data supporting the findings are available within the paper and the Supplementary Materials. Source data used for generating the plots in the main figures are available in the Supplementaty Data file associated with the manuscript. In addition, the original uncropped western images including protein ladders are included in Supplementary Figs. 9-11). Of note, additional information and reagents are available from the corresponding author upon reasonable request.

## References

[1] Chapman, N. M. & Chi, H. Metabolic adaptation of lymphocytes in immunity and disease. *Immunity* **55**, 14–30 (2022). 35021054 10.1016/j.immuni.2021.12.012 PMC8842882

[2] Geltink, R. I. K., Kyle, R. L. & Pearce, E. L. Unraveling the Complex Interplay Between T Cell Metabolism and Function. *Annu Rev. Immunol.* **36**, 461–488 (2018). 29677474 10.1146/annurev-immunol-042617-053019 PMC6323527

[3] Shyer, J. A., Flavell, R. A. & Bailis, W. Metabolic signaling in T cells. *Cell Res.* **30**, 649–659 (2020). 32709897 10.1038/s41422-020-0379-5 PMC7395146

[4] Wik, J. A. & Skalhegg, B. S. T Cell Metabolism in Infection. *Front Immunol.* **13**, 840610 (2022). 35359994 10.3389/fimmu.2022.840610 PMC8964062

[5] Cham, C. M., Driessens, G., O’Keefe, J. P. & Gajewski, T. F. Glucose deprivation inhibits multiple key gene expression events and effector functions in CD8+ T cells. *Eur. J. Immunol.* **38**, 2438–2450 (2008). 18792400 10.1002/eji.200838289 PMC3008428

[6] Cham, C. M. & Gajewski, T. F. Glucose availability regulates IFN-gamma production and p70S6 kinase activation in CD8+ effector T cells. *J. Immunol.* **174**, 4670–4677 (2005). 15814691 10.4049/jimmunol.174.8.4670

[7] Chang, C. H. et al. Posttranscriptional control of T cell effector function by aerobic glycolysis. *Cell* **153**, 1239–1251 (2013). 23746840 10.1016/j.cell.2013.05.016 PMC3804311

[8] Palmer, C. S., Ostrowski, M., Balderson, B., Christian, N. & Crowe, S. M. Glucose metabolism regulates T cell activation, differentiation, and functions. *Front Immunol.* **6**, 1 (2015). 25657648 10.3389/fimmu.2015.00001 PMC4302982

[9] Gubser, P. M. et al. Rapid effector function of memory CD8+ T cells requires an immediate-early glycolytic switch. *Nat. Immunol.* **14**, 1064–1072 (2013). 23955661 10.1038/ni.2687

[10] Menk, A. V. et al. Early TCR Signaling Induces Rapid Aerobic Glycolysis Enabling Distinct Acute T Cell Effector Functions. *Cell Rep.* **22**, 1509–1521 (2018). 29425506 10.1016/j.celrep.2018.01.040 PMC5973810

[11] Angin, M. et al. Metabolic plasticity of HIV-specific CD8(+) T cells is associated with enhanced antiviral potential and natural control of HIV-1 infection. *Nat. Metab.* **1**, 704–716 (2019). 32694646 10.1038/s42255-019-0081-4

[12] Gnanaprakasam, J. N. R. et al. Asparagine restriction enhances CD8(+) T cell metabolic fitness and antitumoral functionality through an NRF2-dependent stress response. *Nat. Metab.* **5**, 1423–1439 (2023). 37550596 10.1038/s42255-023-00856-1 PMC10447245

[13] Loucif, H. et al. Autophagy-dependent glutaminolysis drives superior IL21 production in HIV-1-specific CD4 T cells. *Autophagy* **18**, 1256–1273 (2022). 34612140 10.1080/15548627.2021.1972403 PMC9225533

[14] Loucif, H. et al. Lipophagy confers a key metabolic advantage that ensures protective CD8A T-cell responses against HIV-1. *Autophagy* **17**, 3408–3423 (2021). 33459125 10.1080/15548627.2021.1874134 PMC8632342

[15] O’Sullivan, D. et al. Memory CD8(+) T cells use cell-intrinsic lipolysis to support the metabolic programming necessary for development. *Immunity* **41**, 75–88 (2014). 25001241 10.1016/j.immuni.2014.06.005 PMC4120664

[16] Loucif, H. et al. Plasticity in T-cell mitochondrial metabolism: A necessary peacekeeper during the troubled times of persistent HIV-1 infection. *Cytokine Growth Factor Rev.* **55**, 26–36 (2020). 32151523 10.1016/j.cytogfr.2020.02.004

[17] Almeida, J. R. et al. Superior control of HIV-1 replication by CD8+ T cells is reflected by their avidity, polyfunctionality, and clonal turnover. *J. Exp. Med.* **204**, 2473–2485 (2007). 17893201 10.1084/jem.20070784 PMC2118466

[18] Betts, M. R. et al. HIV nonprogressors preferentially maintain highly functional HIV-specific CD8+ T cells. *Blood* **107**, 4781–4789 (2006). 16467198 10.1182/blood-2005-12-4818 PMC1895811

[19] Cubas, R. et al. Reversible Reprogramming of Circulating Memory T Follicular Helper Cell Function during Chronic HIV Infection. *J. Immunol.* **195**, 5625–5636 (2015). 26546609 10.4049/jimmunol.1501524 PMC4670798

[20] Iannello, A. et al. Dynamics and consequences of IL-21 production in HIV-infected individuals: a longitudinal and cross-sectional study. *J. Immunol.* **184**, 114–126 (2010). 19949086 10.4049/jimmunol.0901967

[21] Botbol, Y., Patel, B. & Macian, F. Common gamma-chain cytokine signaling is required for macroautophagy induction during CD4+ T-cell activation. *Autophagy* **11**, 1864–1877 (2015). 26391567 10.1080/15548627.2015.1089374 PMC4824584

[22] Martinez-Reyes, I. & Chandel, N. S. Mitochondrial TCA cycle metabolites control physiology and disease. *Nat. Commun.* **11**, 102 (2020). 31900386 10.1038/s41467-019-13668-3 PMC6941980

[23] Anckar, J. & Sistonen, L. Heat shock factor 1 as a coordinator of stress and developmental pathways. *Adv. Exp. Med Biol.* **594**, 78–88 (2007). 17205677 10.1007/978-0-387-39975-1_8

[24] Kurop, M. K., Huyen, C. M., Kelly, J. H. & Blagg, B. S. J. The heat shock response and small molecule regulators. *Eur. J. Med Chem.* **226**, 113846 (2021). 34563965 10.1016/j.ejmech.2021.113846 PMC8608735

[25] Malik, J. A. & Lone, R. Heat shock proteins with an emphasis on HSP 60. *Mol. Biol. Rep.* **48**, 6959–6969 (2021). 34498161 10.1007/s11033-021-06676-4

[26] Saibil, H. Chaperone machines for protein folding, unfolding and disaggregation. *Nat. Rev. Mol. Cell Biol.* **14**, 630–642 (2013). 24026055 10.1038/nrm3658 PMC4340576

[27] Bie, A. S. et al. An inventory of interactors of the human HSP60/HSP10 chaperonin in the mitochondrial matrix space. *Cell Stress Chaperones* **25**, 407–416 (2020). 32060690 10.1007/s12192-020-01080-6 PMC7192978

[28] Corydon, T. J., Hansen, J., Bross, P. & Jensen, T. G. Down-regulation of Hsp60 expression by RNAi impairs folding of medium-chain acyl-CoA dehydrogenase wild-type and disease-associated proteins. *Mol. Genet Metab.* **85**, 260–270 (2005). 15927499 10.1016/j.ymgme.2005.04.003

[29] Guo, J. et al. HSP60-regulated Mitochondrial Proteostasis and Protein Translation Promote Tumor Growth of Ovarian Cancer. *Sci. Rep.* **9**, 12628 (2019). 31477750 10.1038/s41598-019-48992-7 PMC6718431

[30] Li, N. et al. HSP60 Regulates Lipid Metabolism in Human Ovarian Cancer. *Oxid. Med Cell Longev.* **2021**, 6610529 (2021). 34557266 10.1155/2021/6610529 PMC8452972

[31] Teng, R. et al. HSP60 silencing promotes Warburg-like phenotypes and switches the mitochondrial function from ATP production to biosynthesis in ccRCC cells. *Redox Biol.* **24**, 101218 (2019). 31112866 10.1016/j.redox.2019.101218 PMC6526248

[32] Parker, S. J. et al. Spontaneous hydrolysis and spurious metabolic properties of alpha-ketoglutarate esters. *Nat. Commun.* **12**, 4905 (2021). 34385458 10.1038/s41467-021-25228-9 PMC8361106

[33] Gandhapudi, S. K. et al. Heat shock transcription factor 1 is activated as a consequence of lymphocyte activation and regulates a major proteostasis network in T cells critical for cell division during stress. *J. Immunol.* **191**, 4068–4079 (2013). 24043900 10.4049/jimmunol.1202831 PMC4520533

[34] Albakova, Z., Mangasarova, Y. & Sapozhnikov, A. Impaired Heat Shock Protein Expression in Activated T Cells in B-Cell Lymphoma. *Biomedicines* **10** (2022). 10.3390/biomedicines10112747 PMC9687880 36359267

[35] Dagenais-Lussier, X. et al. Kynurenine Reduces Memory CD4 T-Cell Survival by Interfering with Interleukin-2 Signaling Early during HIV-1 Infection. *J. Virol.* **90**, 7967–7979 (2016). 27356894 10.1128/JVI.00994-16 PMC4988137

[36] Dagenais-Lussier, X. et al. USP18 is a significant driver of memory CD4 T-cell reduced viability caused by type I IFN signaling during primary HIV-1 infection. *PLoS Pathog.* **15**, e1008060 (2019). 31658294 10.1371/journal.ppat.1008060 PMC6837632

[37] Riou, C. et al. Convergence of TCR and cytokine signaling leads to FOXO3a phosphorylation and drives the survival of CD4+ central memory T cells. *J. Exp. Med* **204**, 79–91 (2007). 17190839 10.1084/jem.20061681 PMC2118424

[38] van Grevenynghe, J. et al. Transcription factor FOXO3a controls the persistence of memory CD4(+) T cells during HIV infection. *Nat. Med* **14**, 266–274 (2008). 18311149 10.1038/nm1728

[39] Morita, R. et al. Human blood CXCR5(+)CD4(+) T cells are counterparts of T follicular cells and contain specific subsets that differentially support antibody secretion. *Immunity* **34**, 108–121 (2011). 21215658 10.1016/j.immuni.2010.12.012 PMC3046815

[40] Zander, R. et al. Tfh-cell-derived interleukin 21 sustains effector CD8(+) T cell responses during chronic viral infection. *Immunity* **55**, 475–493 e475 (2022). 35216666 10.1016/j.immuni.2022.01.018 PMC8916994

[41] Hu, C. et al. Heat shock proteins: Biological functions, pathological roles, and therapeutic opportunities. *MedComm (2020)* **3**, e161 (2022). 35928554 10.1002/mco2.161 PMC9345296

[42] Sharma, C. & Seo, Y. H. Small Molecule Inhibitors of HSF1-Activated Pathways as Potential Next-Generation Anticancer Therapeutics. *Molecules* **23** (2018). 10.3390/molecules23112757 PMC6278446 30356024

[43] Lim, S. A., Su, W., Chapman, N. M. & Chi, H. Lipid metabolism in T cell signaling and function. *Nat. Chem. Biol.* **18**, 470–481 (2022). 35484263 10.1038/s41589-022-01017-3 PMC11103273

[44] Lochner, M., Berod, L. & Sparwasser, T. Fatty acid metabolism in the regulation of T cell function. *Trends Immunol.* **36**, 81–91 (2015). 25592731 10.1016/j.it.2014.12.005

[45] MacIver, N. J., Michalek, R. D. & Rathmell, J. C. Metabolic regulation of T lymphocytes. *Annu Rev. Immunol.* **31**, 259–283 (2013). 23298210 10.1146/annurev-immunol-032712-095956 PMC3606674

[46] Legendre, F., MacLean, A., Appanna, V. P. & Appanna, V. D. Biochemical pathways to alpha-ketoglutarate, a multi-faceted metabolite. *World J. Microbiol Biotechnol.* **36**, 123 (2020). 32686016 10.1007/s11274-020-02900-8

[47] Wu, N. et al. Alpha-Ketoglutarate: Physiological Functions and Applications. *Biomol. Ther. (Seoul.)* **24**, 1–8 (2016). 26759695 10.4062/biomolther.2015.078 PMC4703346

[48] Klysz, D. et al. Glutamine-dependent alpha-ketoglutarate production regulates the balance between T helper 1 cell and regulatory T cell generation. *Sci. Signal* **8**, ra97 (2015). 26420908 10.1126/scisignal.aab2610

[49] Matias, M. I. et al. Regulatory T cell differentiation is controlled by alphaKG-induced alterations in mitochondrial metabolism and lipid homeostasis. *Cell Rep.* **37**, 109911 (2021). 34731632 10.1016/j.celrep.2021.109911 PMC10167917

[50] Araujo, L., Khim, P., Mkhikian, H., Mortales, C. L. & Demetriou, M. Glycolysis and glutaminolysis cooperatively control T cell function by limiting metabolite supply to N-glycosylation. *Elife* **6** (2017). 10.7554/eLife.21330 PMC5257256 28059703

[51] Carr, E. L. et al. Glutamine uptake and metabolism are coordinately regulated by ERK/MAPK during T lymphocyte activation. *J. Immunol.* **185**, 1037–1044 (2010). 20554958 10.4049/jimmunol.0903586 PMC2897897

[52] Corrado, M. & Pearce, E. L. Targeting memory T cell metabolism to improve immunity. *J Clin Invest* **132** (2022). 10.1172/JCI148546 PMC8718135 34981777

[53] Pearce, E. L. et al. Enhancing CD8 T-cell memory by modulating fatty acid metabolism. *Nature* **460**, 103–107 (2009). 19494812 10.1038/nature08097 PMC2803086

[54] Cao, J. et al. Effects of altered glycolysis levels on CD8(+) T cell activation and function. *Cell Death Dis.* **14**, 407 (2023). 37422501 10.1038/s41419-023-05937-3 PMC10329707

[55] Maciver, N. J. et al. Glucose metabolism in lymphocytes is a regulated process with significant effects on immune cell function and survival. *J. Leukoc. Biol.* **84**, 949–957 (2008). 18577716 10.1189/jlb.0108024 PMC2638731

[56] Wofford, J. A., Wieman, H. L., Jacobs, S. R., Zhao, Y. & Rathmell, J. C. IL-7 promotes Glut1 trafficking and glucose uptake via STAT5-mediated activation of Akt to support T-cell survival. *Blood* **111**, 2101–2111 (2008). 18042802 10.1182/blood-2007-06-096297 PMC2234050

[57] Ghahari, N. et al. Harnessing Autophagy to Overcome Antigen-Specific T-Cell Dysfunction: Implication for People Living with HIV-1. *Int. J. Mol. Sci.* **24** (2023). 10.3390/ijms241311018 PMC10342174 37446195

[58] Arnold, P. K. & Finley, L. W. S. Regulation and function of the mammalian tricarboxylic acid cycle. *J. Biol. Chem.* **299**, 102838 (2023). 36581208 10.1016/j.jbc.2022.102838 PMC9871338

[59] Lu, Z. et al. Prolonged fasting identifies heat shock protein 10 as a Sirtuin 3 substrate: elucidating a new mechanism linking mitochondrial protein acetylation to fatty acid oxidation enzyme folding and function. *J. Biol. Chem.* **290**, 2466–2476 (2015). 25505263 10.1074/jbc.M114.606228 PMC4303695

[60] Tang, H. et al. Isotope tracing assisted metabolic profiling: Application to understanding HSP60 silencing mediated tumor progression. *Anal. Chim. Acta* **1047**, 93–103 (2019). 30567669 10.1016/j.aca.2018.09.067

[61] Wu, X. et al. The 60-kDa heat shock protein regulates energy rearrangement and protein synthesis to promote proliferation of multiple myeloma cells. *Br. J. Haematol.* **190**, 741–752 (2020). 32155663 10.1111/bjh.16569

[62] Zhou, C. et al. Oncogenic HSP60 regulates mitochondrial oxidative phosphorylation to support Erk1/2 activation during pancreatic cancer cell growth. *Cell Death Dis.* **9**, 161 (2018). 29415987 10.1038/s41419-017-0196-z PMC5833694

[63] Guo, J., Zhu, S., Deng, H. & Xu, R. HSP60-knockdown suppresses proliferation in colorectal cancer cells via activating the adenine/AMPK/mTOR signaling pathway. *Oncol. Lett.* **22**, 630 (2021). 34267822 10.3892/ol.2021.12891 PMC8258614

[64] Tang, H. et al. Downregulation of HSP60 disrupts mitochondrial proteostasis to promote tumorigenesis and progression in clear cell renal cell carcinoma. *Oncotarget* **7**, 38822–38834 (2016). 27246978 10.18632/oncotarget.9615 PMC5122432

[65] Miller, K. D. et al. Acetate acts as a metabolic immunomodulator by bolstering T-cell effector function and potentiating antitumor immunity in breast cancer. *Nat. Cancer* **4**, 1491–1507 (2023). 37723305 10.1038/s43018-023-00636-6 PMC10615731

[66] Trompette, A. et al. Dietary Fiber Confers Protection against Flu by Shaping Ly6c(-) Patrolling Monocyte Hematopoiesis and CD8(+) T Cell Metabolism. *Immunity* **48**, 992–1005 e1008 (2018). 29768180 10.1016/j.immuni.2018.04.022

[67] Asao, H. Interleukin-21 in Viral Infections. *Int. J. Mol. Sci.* **22** (2021). 10.3390/ijms22179521 PMC8430989 34502427

[68] Woolard, S. N. & Kumaraguru, U. Viral vaccines and CTL response. *J. Biomed. Biotechnol.* **2010**, 141657 (2010). 20379365 10.1155/2010/141657 PMC2850151

[69] Migueles, S. A. et al. Defective human immunodeficiency virus-specific CD8+ T-cell polyfunctionality, proliferation, and cytotoxicity are not restored by antiretroviral therapy. *J. Virol.* **83**, 11876–11889 (2009). 19726501 10.1128/JVI.01153-09 PMC2772718

[70] Mu, W. et al. Autophagy inducer rapamycin treatment reduces IFN-I-mediated Inflammation and improves anti-HIV-1 T cell response in vivo. *JCI Insight* **7** (2022). 10.1172/jci.insight.159136 PMC9746825 36509289

[71] de Armas, L. R. et al. Induction of IL-21 in Peripheral T Follicular Helper cells is Indicator of Influenza Vaccine Response in Previously Vaccinated HIV-Infected Pediatric Cohort. *J. Immunol.* **198**, 1995–2005 (2017). 28130496 10.4049/jimmunol.1601425 PMC5322168

[72] Kvistad, D. et al. IL-21 enhances influenza vaccine responses in aged macaques with suppressed SIV infection. *JCI Insight.* **6** (2021). 10.1172/jci.insight.150888 PMC8564910 34491910

[73] McElhaney, J. E. et al. Key Determinants of Cell-Mediated Immune Responses: A Randomized Trial of High Dose Vs. Standard Dose Split-Virus Influenza Vaccine in Older Adults. *Front. Aging* **2** (2021). 10.3389/fragi.2021.649110 PMC8813165 35128529

[74] Spensieri, F. et al. Human circulating influenza-CD4+ ICOS1+IL-21+ T cells expand after vaccination, exert helper function, and predict antibody responses. *Proc. Natl Acad. Sci. USA* **110**, 14330–14335 (2013). 23940329 10.1073/pnas.1311998110 PMC3761599

[75] Currier, J. R. et al. A panel of MHC class I restricted viral peptides for use as a quality control for vaccine trial ELISPOT assays. *J. Immunol. Methods* **260**, 157–172 (2002). 11792386 10.1016/s0022-1759(01)00535-x

[76] Gomez-Perosanz, M. et al. Characterization of Conserved and Promiscuous Human Rhinovirus CD4 T Cell Epitopes. *Cells* **10** (2021). 10.3390/cells10092294 PMC8471592 34571943

[77] Olagnier, D. et al. HTLV-1 Tax-mediated inhibition of FOXO3a activity is critical for the persistence of terminally differentiated CD4+ T cells. *PLoS Pathog.* **10**, e1004575 (2014). 25521510 10.1371/journal.ppat.1004575 PMC4270795

[78] Sze, A. et al. Host restriction factor SAMHD1 limits human T cell leukemia virus type 1 infection of monocytes via STING-mediated apoptosis. *Cell Host Microbe* **14**, 422–434 (2013). 24139400 10.1016/j.chom.2013.09.009

[79] van Grevenynghe, J. et al. Loss of memory B cells during chronic HIV infection is driven by Foxo3a- and TRAIL-mediated apoptosis. *J. Clin. Invest* **121**, 3877–3888 (2011). 21926463 10.1172/JCI59211 PMC3195482

