# Supplementary material for: HSP60 controls mitochondrial ATP generation for optimal virus-specific IL-21-producing CD4 and cytotoxic CD8 memory T cell responses
Source: Commun Biol. 2024 Dec 21;7:1688. doi: 10.1038/s42003-024-07326-8 (PMC11663223; doi:10.1038/s42003-024-07326-8)
Supplement: Supplementary file 2 — Supplementary Material [file 42003_2024_7326_MOESM2_ESM.pdf]

. HSP60 assessment

	Immunogen	clone ID	fluorophore	vendor
Surface	CD3	UCHT-1	Alexa Fluor 700	BD Biosciences
	CD4	RPA-T4	BV421	BD Biosciences
	CD8	RPA-T8	BV650	BD Biosciences
	CD45RA	HI100	APC H7	BD Biosciences
	CCR7	150503	V450	BD Biosciences
	CD27	L128	FITC	BD Biosciences
	CXCR5	RF8B2	PerCP-Cy5.5	BD Biosciences
	CXCR3	1C6	PE-Cy7	BD Biosciences
ICS	HSP60	D6F1	PE	Cell Signaling
	IFNγ	4S.B3	Alexa Fluor 647	BD Biosciences

. HSF1 assessment [total and pS326 protein forms]

	Immunogen	clone ID	fluorophore	vendor
Surface	CD3	UCHT-1	Alexa Fluor 700	BD Biosciences
	CD4	RPA-T4	BV421	BD Biosciences
	CD8	RPA-T8	BV650	BD Biosciences
	CD45RA	HI100	APC H7	BD Biosciences
ICS	HSF1	D3L8I	PE	Cell signaling
	HSF1 pS326	SU31-03	unconjugated*	In vitrogen (ThermoFisher)
	IFNγ	4S.B3	Alexa Fluor 647	BD Biosciences

*Abs were conjugated to AlexaFluor 488 dye using the Zenon rabbit IgG labeling kit (Life Technologies Inc.)

. Assessment of effector cytokines [IFN-γ, IL-2, and TNF-?]

	Immunogen	clone ID	fluorophore	vendor
Surface	CD3	UCHT-1	Alexa Fluor 700	BD Biosciences
	CD4	RPA-T4	BV421	BD Biosciences
	CD8	RPA-T8	BV650	BD Biosciences
	CD45RA	HI100	APC H7	BD Biosciences
ICS	IFNγ	4S.B3	Alexa Fluor 647	BD Biosciences
	IL-2	MQ1-17H12	FITC	BD Biosciences
	TNF-?	MAb11	BV711	BD Biosciences

. IL-21 assessment on CD4

	Immunogen	clone ID	fluorophore	vendor
Surface	CD3	UCHT-1	Alexa Fluor 700	BD Biosciences
	CD4	RPA-T4	BV421	BD Biosciences
	CD45RA	HI100	APC H7	BD Biosciences
ICS	IL-21	3A3-N2	PE	BD Biosciences
	IFNγ	4S.B3	Alexa Fluor 647	BD Biosciences

. Assessment of cytotoxic molecules on CD8

	Immunogen	clone ID	fluorophore	vendor
Surface	CD3	UCHT-1	Alexa Fluor 700	BD Biosciences
	CD8	RPA-T8	BV650	BD Biosciences
	CD45RA	HI100	APC H7	BD Biosciences
ICS	perforin	B-D48	PerCP-Cy5.5	Biolegend
	Granzyme-B	GB11	PE-CF594	BD Biosciences
	IFNγ	4S.B3	Alexa Fluor 647	BD Biosciences

. Autophagy assessment

	Immunogen	clone ID	fluorophore	vendor
Surface	CD3	UCHT-1	Alexa Fluor 700	BD Biosciences
	CD4	RPA-T4	BV421	BD Biosciences
	CD8	RPA-T8	BV650	BD Biosciences
	CD45RA	HI100	APC H7	BD Biosciences
ICS	ATG1	F-4	PE	Santa Cruz Biotechnology
	Beclin-1	EPR 20473	Alexa Fluor 647	Abcam

Supplementary Table 1. **List of all monoclonal Abs used**, including the information about the vendor, clone, and fluorophore as well as our Ab cocktail to characterize HSF1, HSP60, T cell effector functions and autophagy in gated 7-AAD^{neg}CD4 and CD8 TM, including IFN-γ^{+} virus-specific cells.

Figure S1

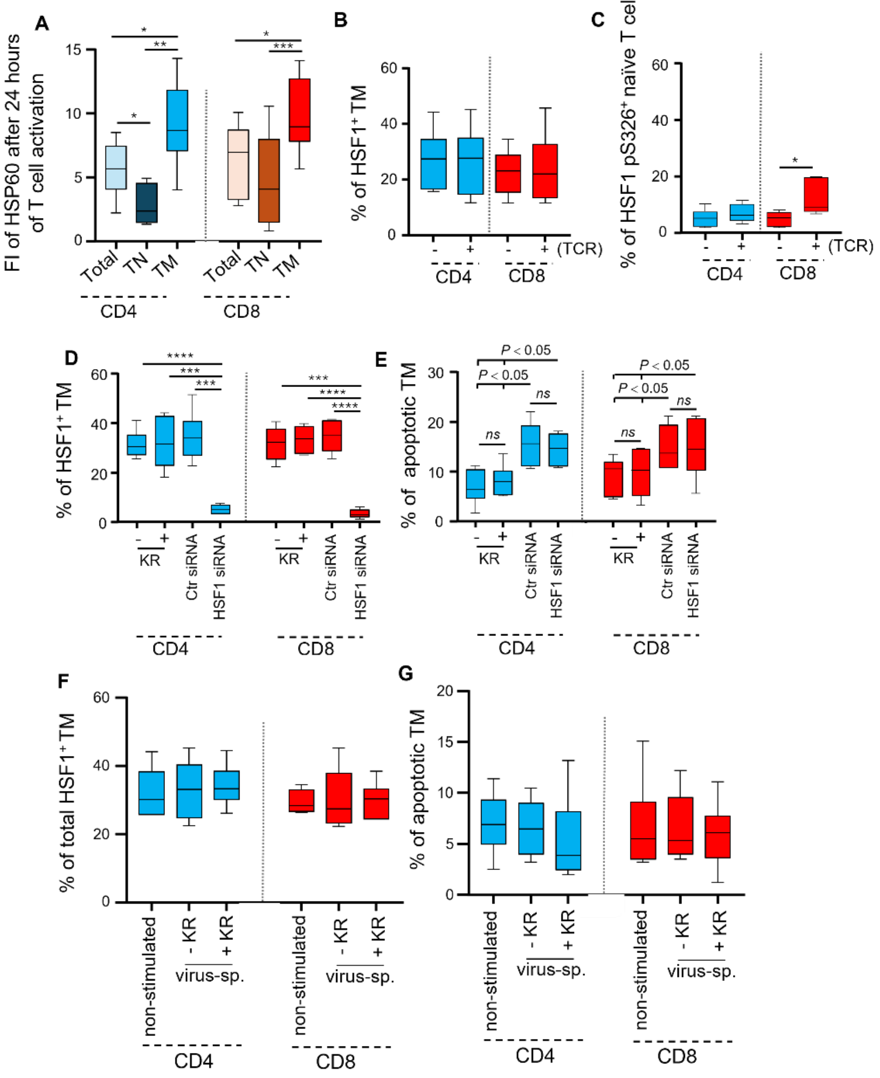

Supplementary Figure 1. **Additional flow assessments in CD4 and CD8 T cells, including virus-specific TM, at 24 hours post-activation** [related to Figs. 1 and 2]. **(A-C)** Briefly, PBMC were TCR-engaged with anti-CD3/CD28 Abs for 24 hours or were not. **(A)** Fold increases (FI) of HSP60 expression in activated CD4 and CD8 TN and TM at 24 hours of culture; FI = [% of HSP60$^+$ activated T cells] / [% of HSP60$^+$ non-activated T cells]. **(B)** % of HSF1$^+$ CD4 and CD8 TM, and **(C)** % of HSF-1 pS326+ naïve CD4 and CD8 T cells at 24 hours of culture w/wo TCR engagement. **(D, E)** Purified CD4 and CD8 TM were pre-transfected with Ctr siRNA or HSF1 siRNA or were not, after which they were cultivated w/wo anti-CD3/CD28 Abs for 24 hours w/wo KR. **(D)** % of HSF1$^+$ and **(E)** apoptotic CD4 and CD8 TM at 24 hours of culture. Ns, non-significant. As expected, cell transfection led to 5-8% increase in levels of TM apoptosis when compared to non-electroporated cells. **(F, G)** PBMC were stimulated or not with viral peptides and anti-CD28 Abs for 24 hours w/wo KR. **(F)** % of HSF1$^+$ and **(G)** Annexin-V$^+$ virus-specific (virus-sp.) CD4 and CD8 TM, and non-stimulated cells at 24 hours of culture. **(A-G)** Data shown are expressed as mean ± SD of n = 6.

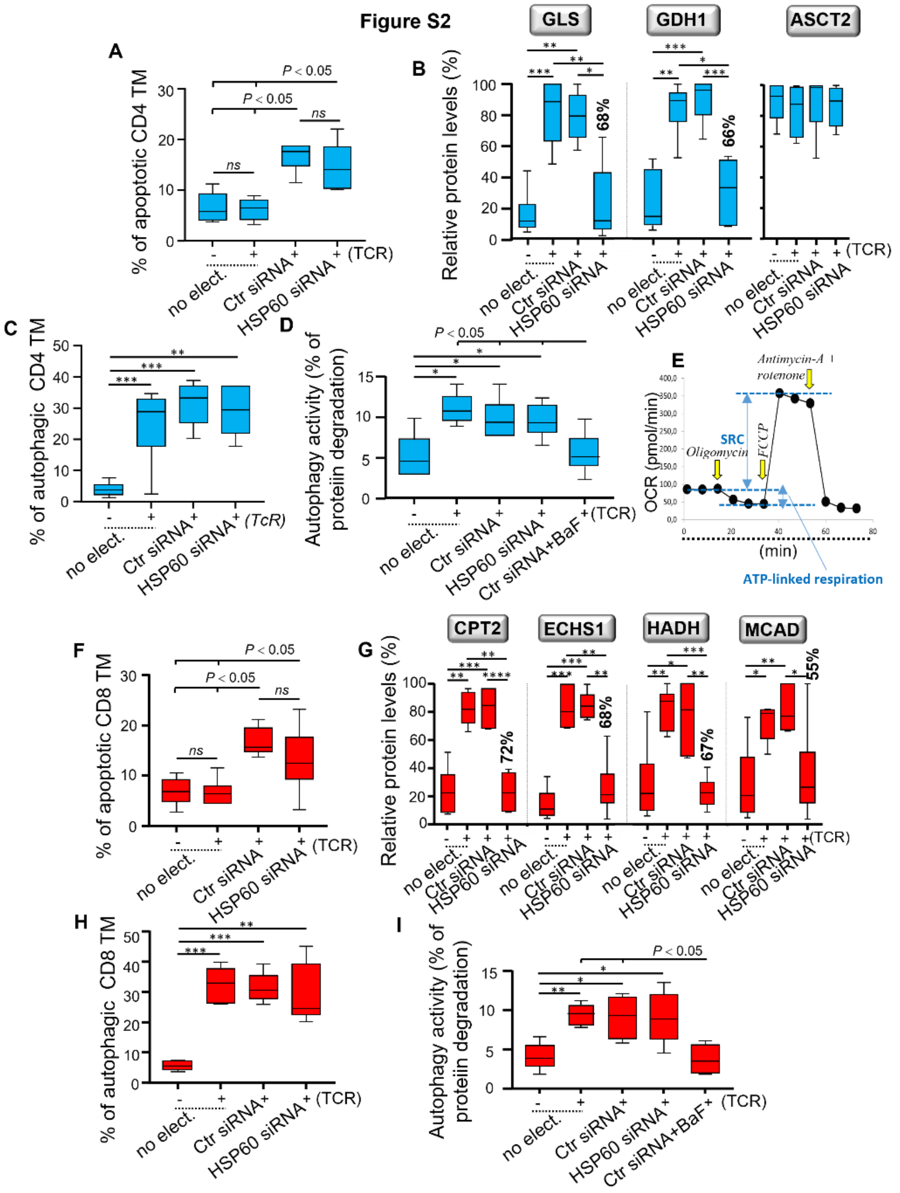

Supplementary Figure 2. **Additional data regarding specific *Hspd1* gene silencing in activated CD4 and CD8 TM** [related to Figs. 3 and 4]. **(A-I)** Briefly, purified CD4 and CD8 TM were pre-transfected with Ctr siRNA or HSP60 siRNA or were not, and then cultivated w/wo anti-CD3/CD28 Abs for 24 hours w/wo BaF. **(A)** % of apoptotic CD4 TM at 24 hours of culture. Of note, cell transfection led to 5-8% expected increase in levels of TM apoptosis when compared to non-electroporated cells. Ns, non-significant. **(B)** Relative protein levels of GLS, GDH1 and ASCT2 transporter determined with Image J software [after normalization with β-actin]. Inhibition % of HSP60 protein levels through specific *Hspd1* gene silencing are also indicated in bold. **(C)** % of autophagic ATG-1^{+}Beclin-1^{+} CD4 TM. **(D)** Autophagy lytic activity using a pulse-chase method. The BaF-mediated inhibition of lysosomal-dependent autophagy activity was also conducted on Ctr siRNA-transfected cells as experimental control. **(E)** Representative *Seahorse* kinetic to show how SRC and ATP-linked respiration values are determined. (**F)** % of apoptotic CD8 TM w/wo specific *Hspd1* gene silencing. **(G)** Relative protein levels of CPT2, ECHS1, HADH and MCAD determined with Image J software (after normalization with β-actin). **(H)** % of autophagic ATG-1^{+}Beclin-1^{+} CD8 TM and their **(I)** Autophagy lytic activity at 24 hours of culture. **(A-I)** Data are expressed as mean ± SD of n = 6.

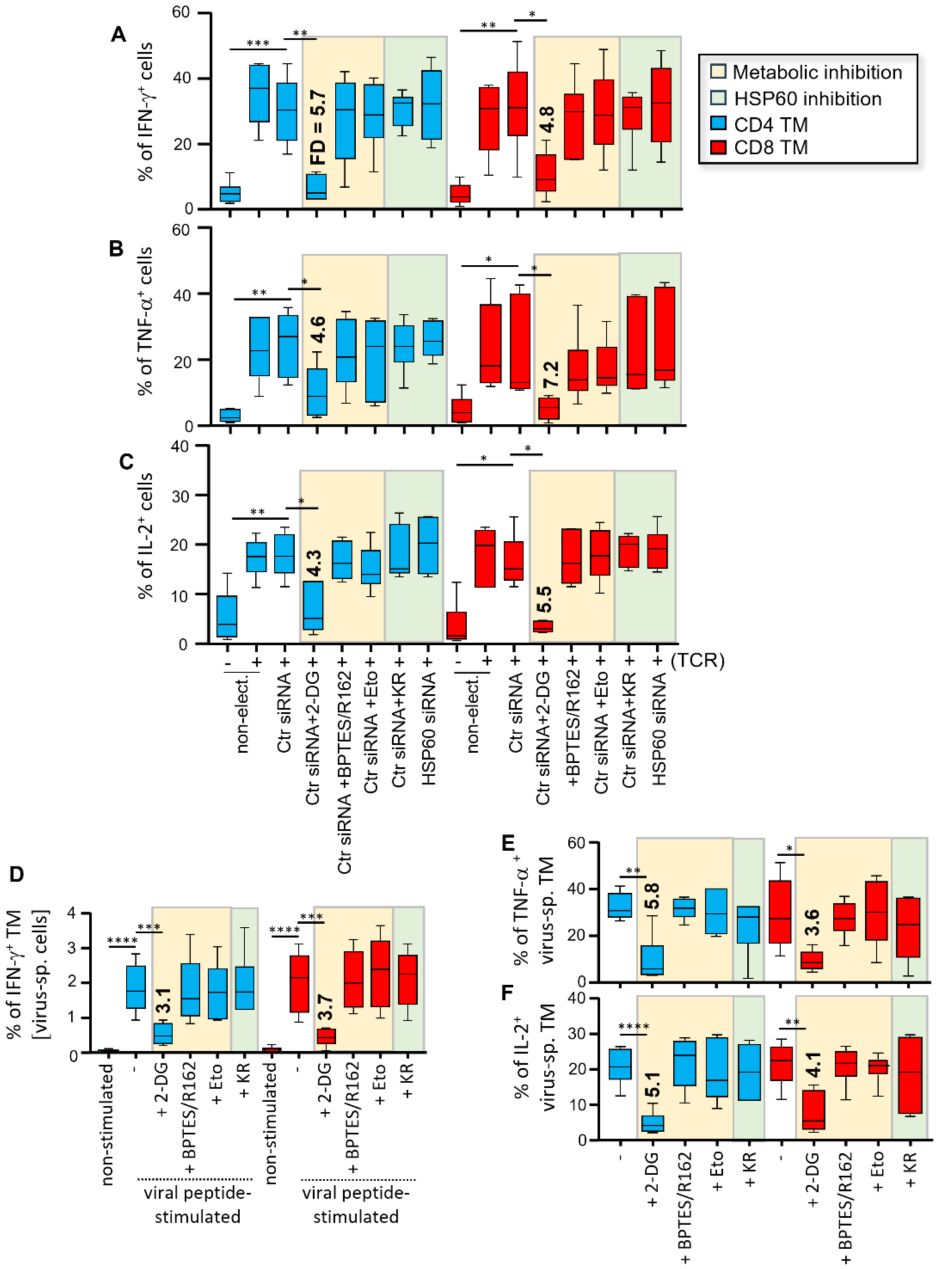

Supplementary Figure 3. **Glycolysis-dependent IFN-γ, TNF-α and IL-2 production in activated TM, including virus-specific cells, are not affected by HSP60 inhibition. (A-C)** CD4 and CD8 TM were pre-transfected with Ctr siRNA or HSP60 siRNA or were not, and then cultivated w/wo anti-CD3/CD28 Abs for 24 hours w/wo KR, 2-DG, BPTES/R162 and Eto. **(A)** % of IFN-γ^+, **(B)** TNF-α^+, and **(C)** IL-2^+ CD4 and CD8 TM at 24 hours of culture. Fold decreases (FD) of cytokine expression are also indicated in bold. Of note, statistical analyses have been conducted for each study condition only when compared to activated Ctr siRNA-transfected TM. **(D-F)** Briefly, PBMC were stimulated or not with viral peptides for 24 hours w/wo KR and metabolic inhibitors [2-DG, BPTES/R162 and Eto]. **(D)** % IFN-γ^+ cells [virus-specific cells] in CD4 and CD8 TM at 24 hours of culture w/wo peptide stimulation. **(E)** % of TNF-α^+ and **(F)** IL-2^+ virus-specific TM at 24 hours post-stimulation. FD of virus-specific T cell effector functions are also indicated in bold. Of note, 3.10^6 PBMC were used for assessing TNF-α and IL-2 in virus-specific TM when they were stimulated with 2-DG. **(A-F)** Results are the mean ± SD of n = 6.

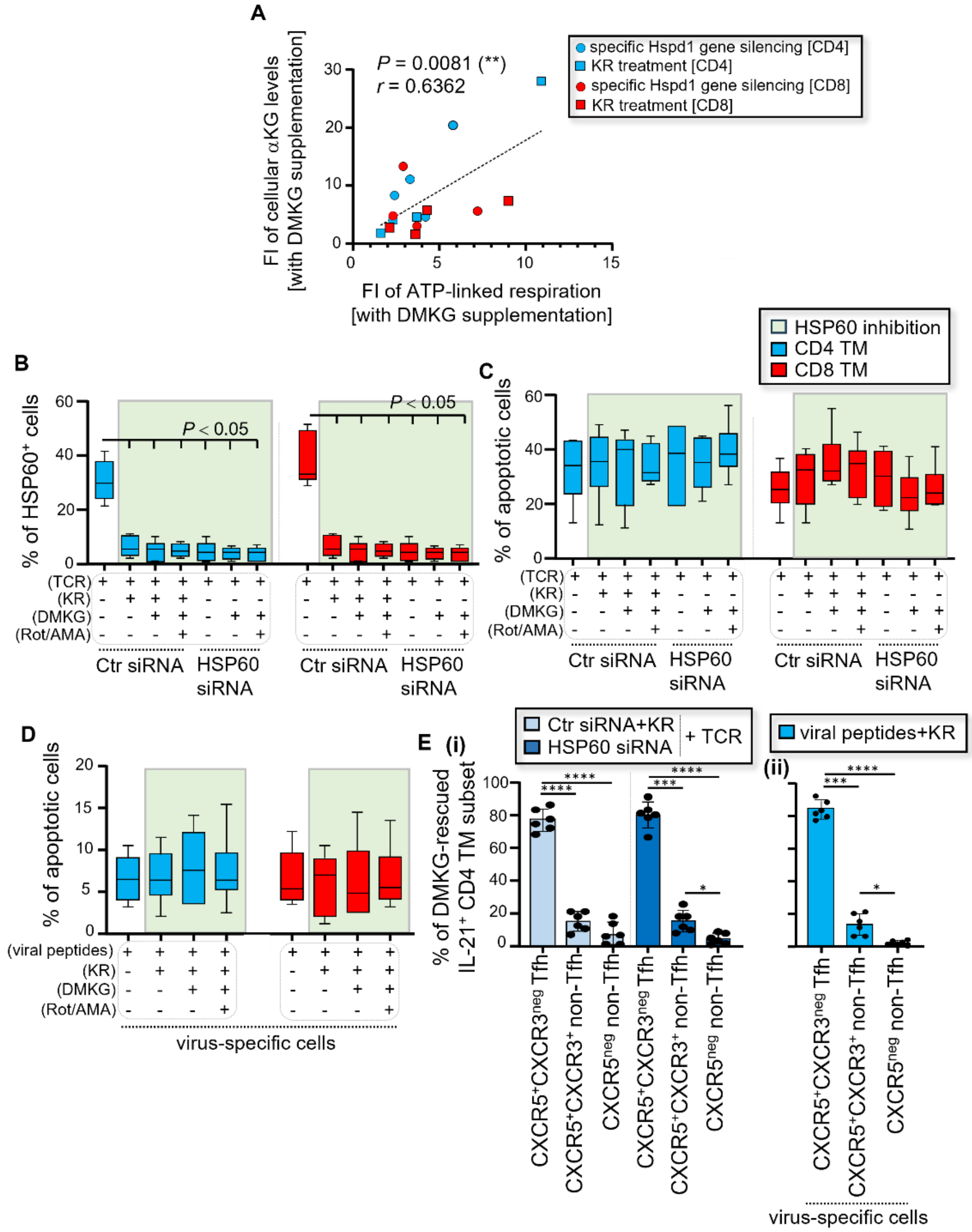

Supplementary Figure 4. **Additional data regarding DMKG supplementation on effector CD4 and CD8 TM.** [related to Fig. 8, except for A]. **(A)** Correlation between fold increases (FI) of ATP-linked respiration and those of cellular αKG levels in HSP60-depleted TM, when cells were activated with DMKG. N = 16 CD4 and CD8 TM w/wo specific *Hspd1* gene silencing and KR at 24 hours post-activation. **(B, C)** Purified CD4 and CD8 TM were pre-transfected with Ctr siRNA or HSP60 siRNA, after which they were T cell activated with anti-CD3/CD28 Abs for 24 hours w/wo KR, DMKG and Rot/AMA. Assessment at 24 hours post-activation of **(B)** their intracellular HSP60 expression along with the **(C)** % of apoptotic cells. Ns, non-significant. **(D)** PBMC were stimulated by viral peptides w/wo KR, DMKG and Rot/AMA. At 24 hours of peptide stimulation, levels of cell apoptosis were determined in virus-specific CD4 and CD8 TM. **(E)** Proportion of Tfh and non-Tfh cells within IL-21-producing CD4 TM when cells were T cell activated in the presence of HSP60 inhibition and rescued by DMKG supplementation; **(i)** TCR-engagement with anti-CD3/CD28 Abs and **(ii)** stimulation with viral peptides for 24 hours. **(B-E)** Data shown are the mean ± SD of n = 6.

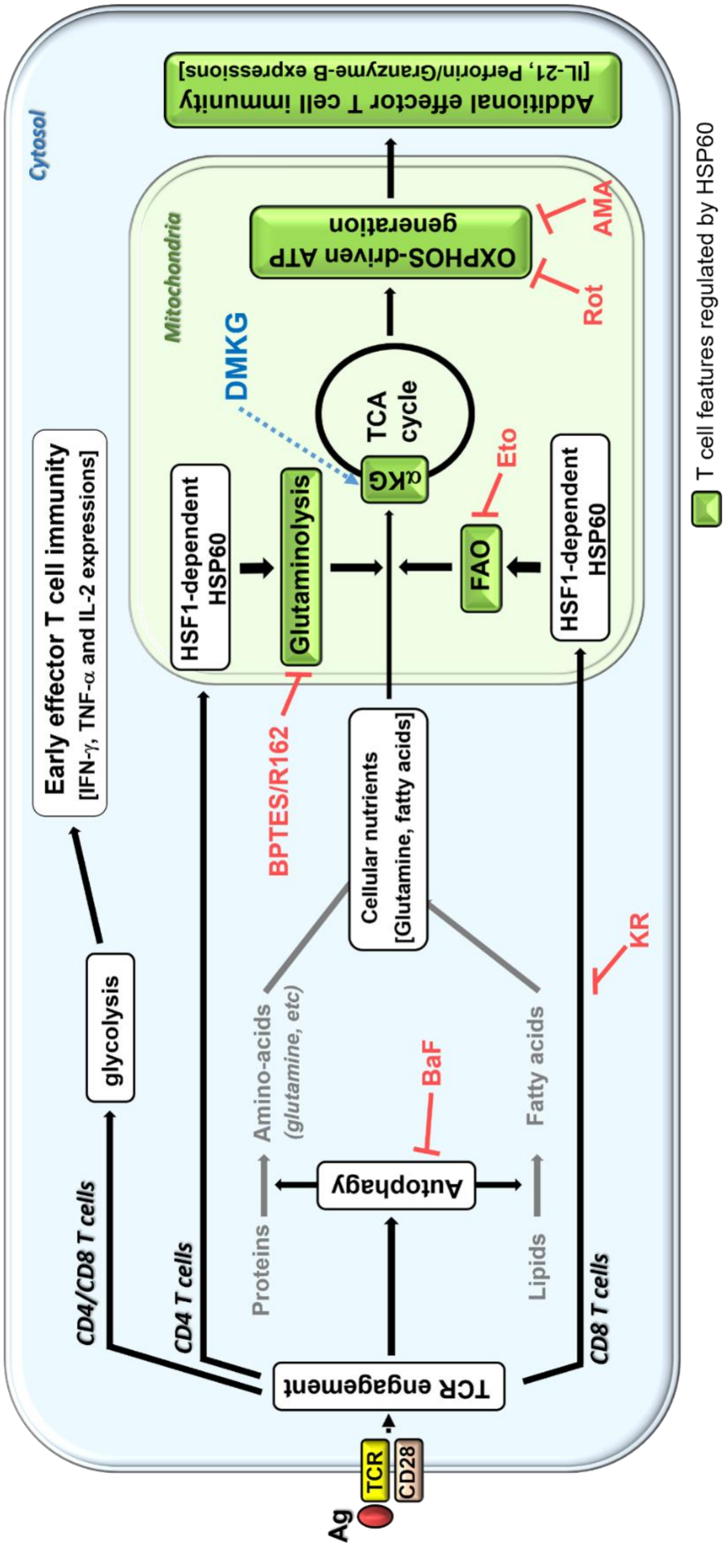

Supplementary Figure 5. **Metabolic rewiring in virus-specific CD4 and CD8 T cells to produce pro-inflammatory cytokines [glycolysis] and, IL-21 and cytotoxic molecules [OXPHOS].** The schematic includes HSP60 as a key player of mitochondrial ATP generation by bolstering both glutaminolytic and FAO pathways after TCR engagement. Of note, DMKG, KR, and all metabolic inhibitors [BPTES, R162, Eto, Rot, AMA, and BaF] are also indicated in red.

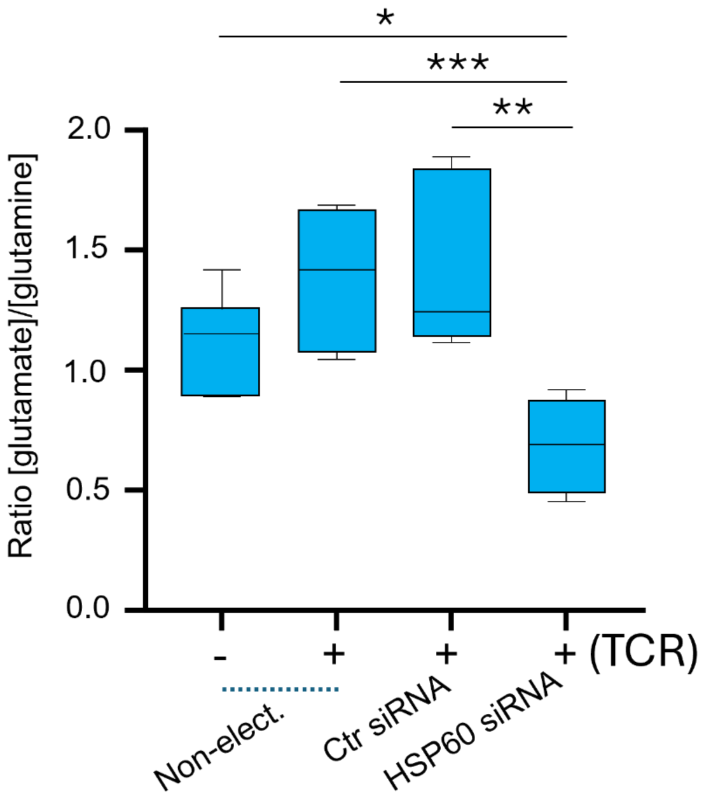

Supplementary Figure 6. **HSP60 inhibition in activated CD4 TM leads to lower glutamate/glutamine ratio [indicative value of reduced glutamine consumption].** Briefly, purified CD4 TM were pre-transfected with Ctr siRNA or HSP60 siRNA or were not, after which they were cultivated w/wo anti-CD3 and CD28 Abs for 24 hours in the presence of R162 drug. Then, cells were collected to assess their cellular concentrations of glutamine and glutamate in uM. Data shown are the ratio [glutamate]/[glutamine] for all study conditions. Fold decrease (FD) in activated CD4 TM when HSP60 was inhibited are also indicated in bold. Mean ± SD of n = 6.

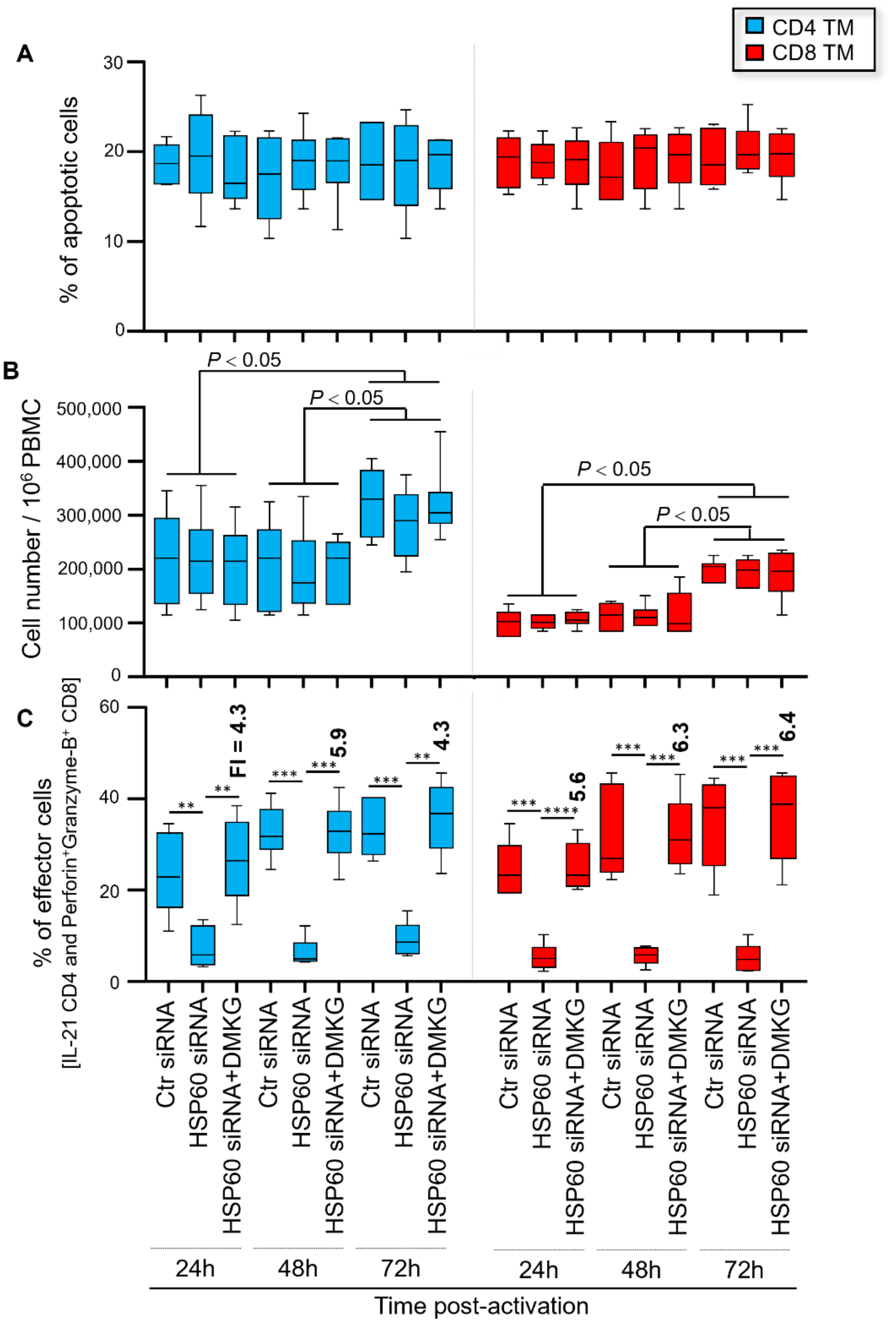

Supplementary Figure 7. **Longer time periods of specific *Hspd1* gene silencing in CD4 and CD8 TM neither influence cell viability/number nor their ability to rescue defective T cell effector functions with DMKG.** Briefly, purified CD4 and CD8 TM were pre-transfected with Ctr siRNA or HSP60 siRNA and then T cell activated with anti-CD3/CD28 Abs for 24-, 48- and 72-hours w/wo DMKG supplementation. At 24-, 48- and 72-hours post-activation, we assessed the **(A)** levels of cell apoptosis, and **(B)** numbers per million PBMC. Of note, TM numbers per 10^6 PBMC were determined as follow: [% of TM in PBMC] * 10^6. **(C)** Assessment of IL-21 production in CD4, and Perforin/Granzyme-B co-expression in CTL TM, when activated w/wo DMKG for 24 to 72 hours. Fold increases (FI) of these effector functions with DMKG supplementation are also indicated in bold. **(A-C)** Data shown are the mean ± SD of n = 6.

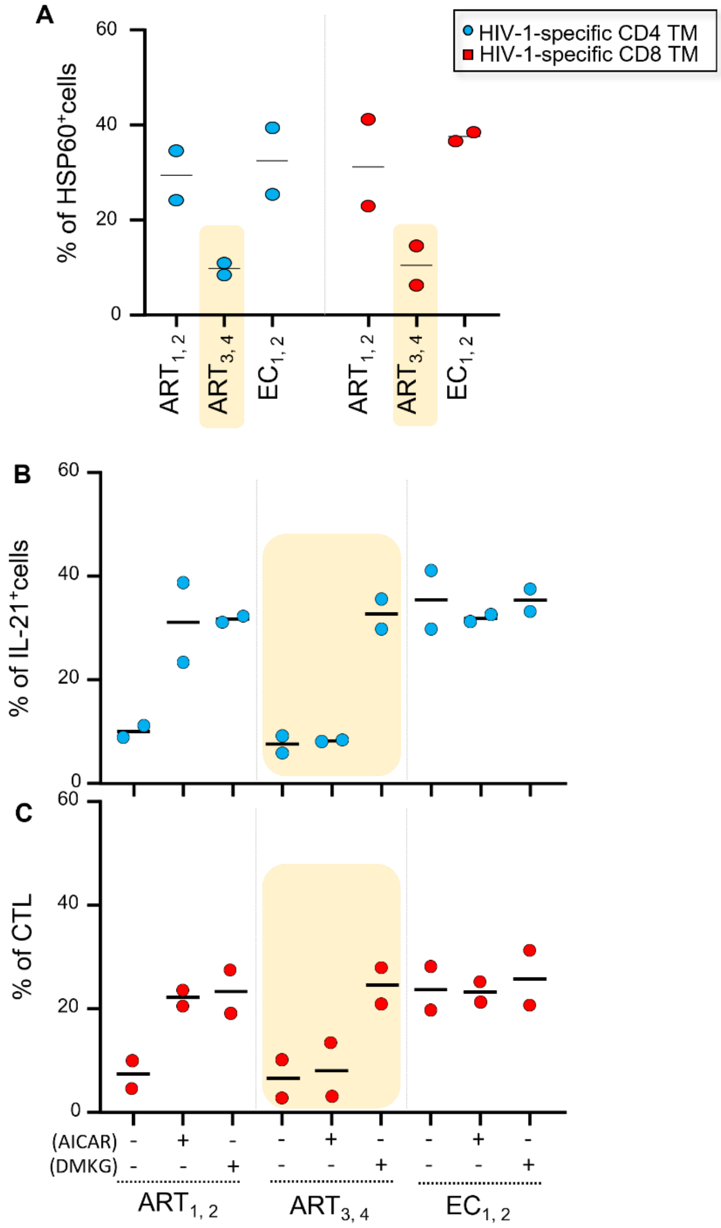

Supplementary Figure 8. **When HSP60 is impaired in HIV-1-specific T cells after TCR engagement, their defective IL-21 and cytotoxic molecule production cannot be rescued by the autophagy inducer AICAR, but rather by DMKG supplementation.** *Of note, we selected four ART-treated and HIV-1-infected individuals ($ART_{1\text{-}4}$) and two EC ($EC_{1,2}$) from our previous works [13,14] [inclusion criteria: age-matched men, presumed HIV-1 infection for a minimum of 4 years, early administration of ART (within first months of primary-infection) for $ART_{1\text{-}4}$ and no ART for $EC_{1,2}$, along with CD4 counts over 400 cells/µl in blood and sustained undetectable viral loads (< 50 copies/ ml) for more than 3 years]. ART_1 and $_2$ responded well to AICAR rescue, while ART_3 and $_4$ did not (shown in yellow).* **(A, B)** Briefly, PBMC were stimulated by 5 µg/mL of HIV-1 p55 (Austral Biologicals) and p24 Gag antigens (Austral Biologicals) with 1 µg/mL anti-CD28 Abs and 10 µM AZT [$ART_{1\text{-}4}$] for 24 hours, w/wo 50µM AICAR (Sigma Aldrich) and DMKG supplementation. **(A)** Levels of intracellular HSP60 expression shown in IFN-γ^+ HIV-1-specific CD4 and CD8 TM at 24 hours post-activation. **(B)** % of HIV-1-specific IL-21$^+$CD4 and **(C)** Perforin$^+$Granzyme-B$^+$CD8 TM (CTL) at 24 hours post-activation w/wo AICAR and DMKG supplementation.

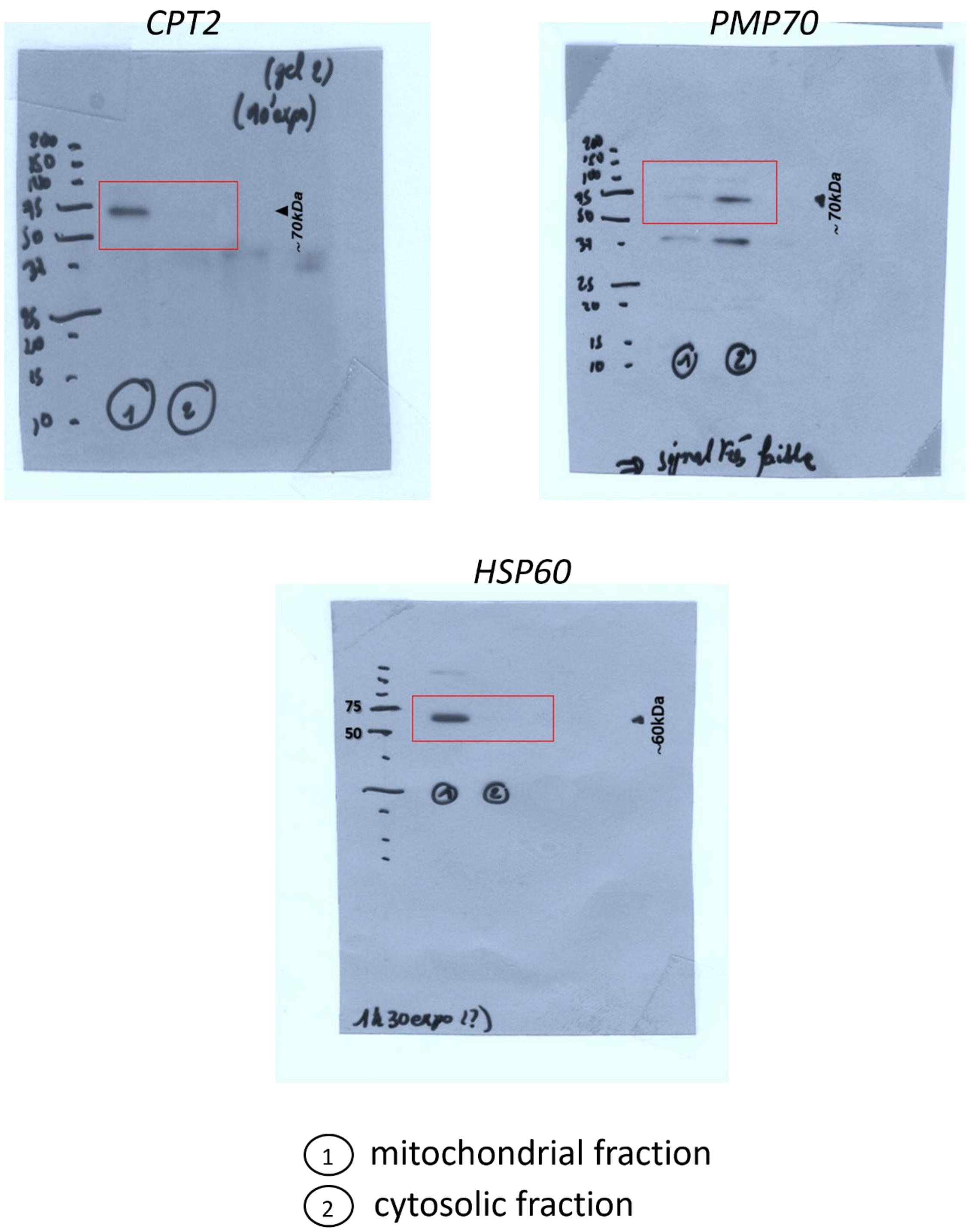

Supplementary Figure 9. **Uncropped western images** [mitochondrial localization of HSP60 in activated Mem; **Fig. 1c**] **including protein ladder.**

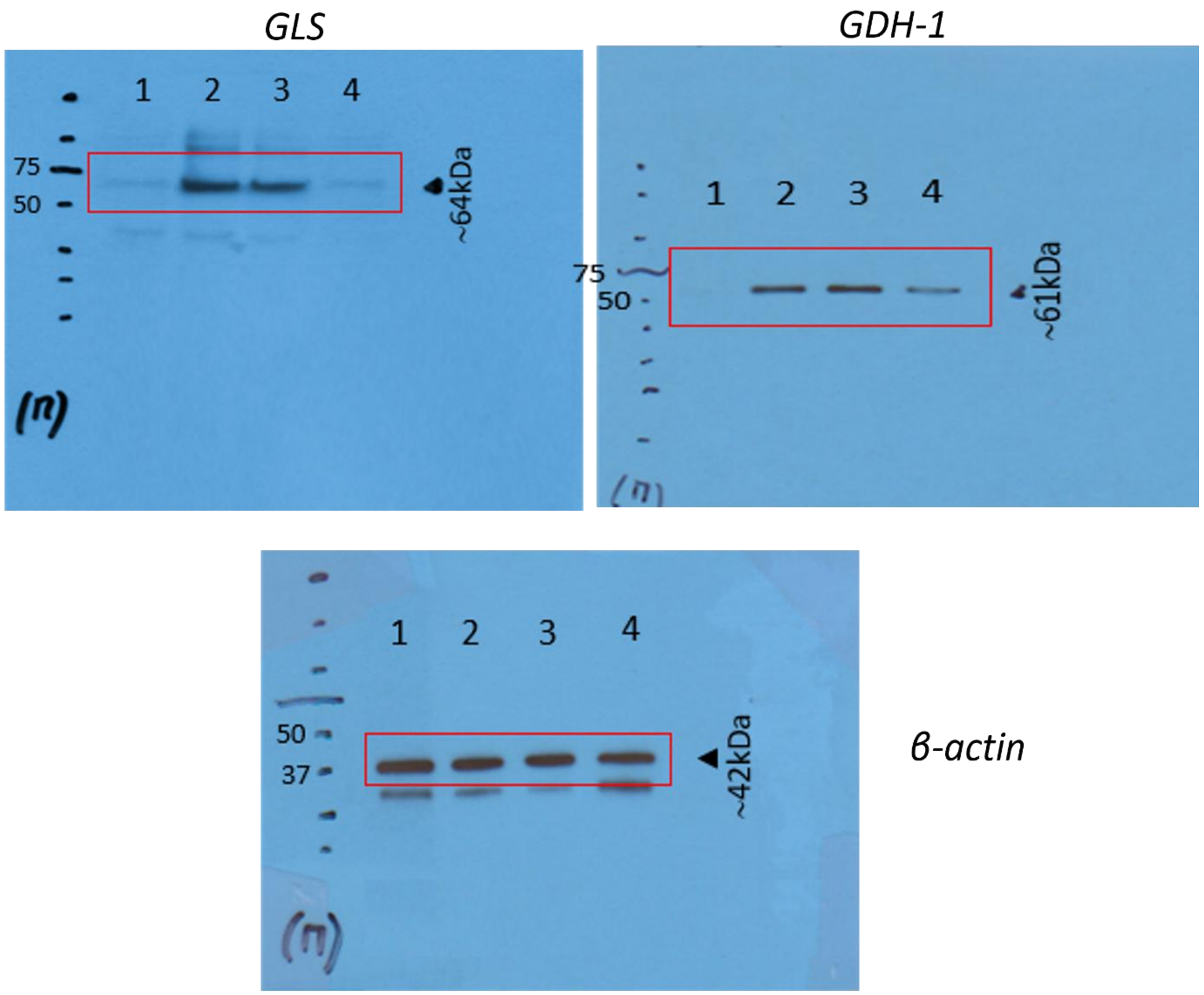

(1) - TcR; no electroporation

(2) + TcR; no electroporation

(3) + TcR; Ctr siRNA

(4) + TcR; HSP_{60} siRNAs

Supplementary Figure 10. **Uncropped western images** [impact of HSP60 silencing on glutaminolytic enzymes in CD4 Mem ± TCR triggering; **Fig. 3c**] **including protein ladder.**

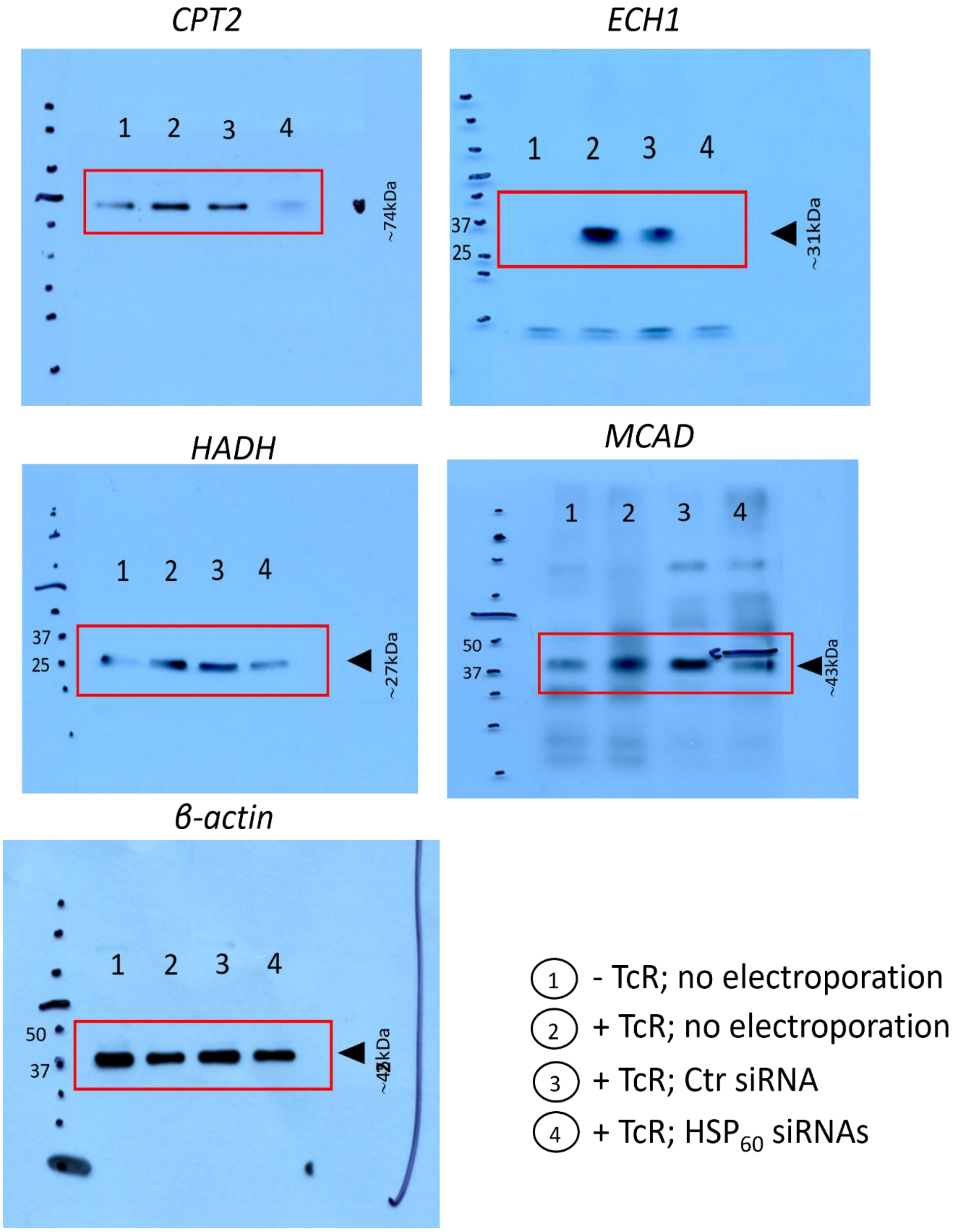

Supplementary Figure 11. **Uncropped western images** [impact of HSP60 silencing on FAO-related enzymes and CPT2 in CD8 Mem ± TCR triggering; **Fig. 4c**] **including protein ladder.**
