# Supplementary material for: HSP60 controls mitochondrial ATP generation for optimal virus-specific IL-21-producing CD4 and cytotoxic CD8 memory T cell responses
Source: Commun Biol. 2024 Dec 21;7:1688. doi: 10.1038/s42003-024-07326-8 (PMC11663223; doi:10.1038/s42003-024-07326-8)
Supplement: Supplementary file 3 — Description of Additional Supplementary Materials [file 42003_2024_7326_MOESM3_ESM.pdf]

Description of Additional Supplementary Files

File name: Supplementary Data

Description: All numerical source data for graphs
