# Supplementary material for: HSP60 controls mitochondrial ATP generation for optimal virus-specific IL-21-producing CD4 and cytotoxic CD8 memory T cell responses
Source: Commun Biol. 2024 Dec 21;7:1688. doi: 10.1038/s42003-024-07326-8 (PMC11663223; doi:10.1038/s42003-024-07326-8)
Supplement: Supplementary file 5 — Reporting Summary [file 42003_2024_7326_MOESM5_ESM.pdf]

nature portfolio

Corresponding author(s): Julien van Grevenynghe

Last updated by author(s): Nov 25, 2024

Reporting Summary

Nature Portfolio wishes to improve the reproducibility of the work that we publish. This form provides structure for consistency and transparency in reporting. For further information on Nature Portfolio policies, see our Editorial Policies and the Editorial Policy Checklist.

Statistics

For all statistical analyses, confirm that the following items are present in the figure legend, table legend, main text, or Methods section.

n/a	Confirmed	
☐	☒	The exact sample size (*n*) for each experimental group/condition, given as a discrete number and unit of measurement
☐	☒	A statement on whether measurements were taken from distinct samples or whether the same sample was measured repeatedly
☐	☒	The statistical test(s) used AND whether they are one- or two-sided<br>*Only common tests should be described solely by name; describe more complex techniques in the Methods section.*
☐	☒	A description of all covariates tested
☐	☒	A description of any assumptions or corrections, such as tests of normality and adjustment for multiple comparisons
☐	☒	A full description of the statistical parameters including central tendency (e.g. means) or other basic estimates (e.g. regression coefficient) AND variation (e.g. standard deviation) or associated estimates of uncertainty (e.g. confidence intervals)
☐	☒	For null hypothesis testing, the test statistic (e.g. *F*, *t*, *r*) with confidence intervals, effect sizes, degrees of freedom and *P* value noted<br>*Give P values as exact values whenever suitable.*
☐	☒	For Bayesian analysis, information on the choice of priors and Markov chain Monte Carlo settings
☐	☒	For hierarchical and complex designs, identification of the appropriate level for tests and full reporting of outcomes
☐	☒	Estimates of effect sizes (e.g. Cohen's *d*, Pearson's *r*), indicating how they were calculated

Our web collection on statistics for biologists contains articles on many of the points above.

Software and code

Policy information about availability of computer code

Data collection	No software used for data collection.
Data analysis	GraphPad Prism 8.4.0

For manuscripts utilizing custom algorithms or software that are central to the research but not yet described in published literature, software must be made available to editors and reviewers. We strongly encourage code deposition in a community repository (e.g. GitHub). See the Nature Portfolio guidelines for submitting code & software for further information.

Data

Policy information about availability of data

All manuscripts must include a data availability statement. This statement should provide the following information, where applicable:

- Accession codes, unique identifiers, or web links for publicly available datasets
- A description of any restrictions on data availability
- For clinical datasets or third party data, please ensure that the statement adheres to our policy

Provide your data availability statement here.

Research involving human participants, their data, or biological material

Policy information about studies with human participants or human data. See also policy information about sex, gender (identity/presentation), and sexual orientation and race, ethnicity and racism.

Reporting on sex and gender	Our Findings apply to only sex paramater (no gender consideration).<br>However, we were not able to collect enough blood samples from female donors (with comparable study criteria: middle-aged participants with no viral infections) to include sex parameter along with robust statistical conclusions; Therefore, our study uses blood sample collections from middle-aged men.
Reporting on race, ethnicity, or other socially relevant groupings	No socially constructed or socially relevant categorization variable(s) used in our manuscript.
Population characteristics	Leukaphereses from healthy subjects (middle-aged men [25 to 50 years old])
Recruitment	Partcipants were all recruited by J.P. Routy (mediacal practicioner) and A. Massicote (nurse), at McGill University Health Centre, Montreal, 633 Quebec, Canada.
Ethics oversight	Each donor signed informed consent forms approved by the McGill 634 University Health Centre research ethics board [ethic number: 2021-7111].

Note that full information on the approval of the study protocol must also be provided in the manuscript.

Field-specific reporting

Please select the one below that is the best fit for your research. If you are not sure, read the appropriate sections before making your selection.

☒ Life sciences ☐ Behavioural & social sciences ☐ Ecological, evolutionary & environmental sciences

For a reference copy of the document with all sections, see nature.com/documents/nr-reporting-summary-flat.pdf

Life sciences study design

All studies must disclose on these points even when the disclosure is negative.

Sample size	Assuming that we would detect more than 10% data difference between two culture conditions, a sample size of 6 individuals per study group have been calculated using the G*power software to ensure a statistical power superior to 90%. Spearman's correlation test was used to identify the association between two variables and we systematically used the two-sided Student paired t test to compare two different in vitro conditions. P values of less than 0.05 were considered as significant difference between two parameters.
Data exclusions	No data were excluded from the analyses.
Replication	When done, all attempts at replication were successfull.
Randomization	All experiments rely on the comparison of metabolic/effector features between two study conditions in PBMCs from middle-aged men (with no infection with HIV-1 and hepatatis viruses).
Blinding	The investigators were blinded during data collection and/or analyses.

Reporting for specific materials, systems and methods

We require information from authors about some types of materials, experimental systems and methods used in many studies. Here, indicate whether each material, system or method listed is relevant to your study. If you are not sure if a list item applies to your research, read the appropriate section before selecting a response.

Materials & experimental systems

n/a	Involved in the study	
☐	☒	Antibodies
☒	☐	Eukaryotic cell lines
☒	☐	Palaeontology and archaeology
☒	☐	Animals and other organisms
☒	☐	Clinical data
☒	☐	Dual use research of concern
☒	☐	Plants

Methods

n/a	Involved in the study	
☒	☐	ChIP-seq
☐	☒	Flow cytometry
☒	☐	MRI-based neuroimaging

Antibodies

Antibodies used	see Table S1 (indications are all provided for the flow cytometry-based antibodies)
Validation	idem

Plants

Seed stocks	n/a
Novel plant genotypes	n/a
Authentication	n/a

Flow Cytometry

Plots

Confirm that:

☒ The axis labels state the marker and fluorochrome used (e.g. CD4-FITC).

☒ The axis scales are clearly visible. Include numbers along axes only for bottom left plot of group (a 'group' is an analysis of identical markers).

☒ All plots are contour plots with outliers or pseudocolor plots.

☒ A numerical value for number of cells or percentage (with statistics) is provided.

Methodology

Sample preparation	1 million of collected PBMCs per study condition
Instrument	BD LSRII Fortessa flow cytometer
Software	DIVA
Cell population abundance	200,000-500,000 gated cells were analyzed for each sample
Gating strategy	shown in several figures and supplemental materials of our manuscript

☒ Tick this box to confirm that a figure exemplifying the gating strategy is provided in the Supplementary Information.
