# Supplementary material for: HSP60 controls mitochondrial ATP generation for optimal virus-specific IL-21-producing CD4 and cytotoxic CD8 memory T cell responses
Source: Commun Biol. 2024 Dec 21;7:1688. doi: 10.1038/s42003-024-07326-8 (PMC11663223; doi:10.1038/s42003-024-07326-8)
Supplement: Supplementary file 1 — Transparent Peer Review file [file 42003_2024_7326_MOESM1_ESM.pdf]

nature portfolio

Peer Review File

HSP60 controls mitochondrial ATP generation for optimal virus-specific IL-21-producing CD4 and cytotoxic CD8 memory T cell responses

Corresponding Author: Dr julien van Grevenynghe

This file contains all reviewer reports in order by version, followed by all author rebuttals in order by version.

Version 0:

Reviewer comments:

Reviewer #1

(Remarks to the Author)
In this manuscript, van Grevenynghe and collaborators aimed to define how mitochondrial ATP promotes the effector function of memory-phenotype CD4 and CD8 T cells, namely IL-21 production by Tfh cells and cytotoxic molecule production by CD8 T cells. Using a series of quantitative and qualitative in vitro approaches, the authors data suggest a crucial role for HSP60, a mitochondria chaperone, in the induction of glutaminolysis and FAO in CD4 and CD8 T memory cells, respectively.

Overall, the study is well-developed, and the experimental design is properly described. Results offer robust evidence for the conclusions of the authors. A few additional experiments would strengthen the conclusions of this paper. More specifically:

1- If possible, the authors should track and compare, in HSP-KD versus WT cells, the glutamine consumption and incorporation into the glutaminolysis pathway, through the use of labeled metabolomics. This would be increased evidence that engagement of glutaminolysis is indeed affected in the absence of HSP. If this experiment (as well as a similar experiment with traced fatty acids for FAO) is technically unfeasible, the authors should discuss the possible future need for such measurements.

2- Importantly, the only rescue experiment for HSP-KD T cells was the administration of an a-KG analog, which provides valid evidence but is not perfect: a-KG can be the byproduct of many different metabolic pathways, being a canonical intermediate of the TCA cycle. A more precise experiment would be the forced expression of glutaminolysis/FAO enzymes in cells treated with KR.

Minor issue:

On line 231, please substitute "Fig. 3D" with "Fig. 3E".

Reviewer #2

(Remarks to the Author)
Through a series of metabolic inhibition and immune based assays the authors show virus specific CD4 and CD8 TM generate mitochondrial ATP through glutaminolysis and FAO pathways, and that these pathways are dependent on HSP60 induction following TCR engagement. Interestingly, they showed that while glycolysis is important early for the production of inflammatory cytokines such is IFN-y, other read-outs of CD4 and CD8 TM cell effector functions such as IL-21 and granzymeB/Perforin production were reliant on HSP60-mediated OXPHOS. This work provides important mechanistic insights in T cell biology, and immunity, and offers new ideas to therapeutically modulate T cell metabolism, with potential clinical benefits. I have a few queries that are generally technical/edits.

1. Authors found increased HSF1 (pS326+) in Tm cells, which correlated with HSP60 expression. Although they found the greatest expression of HSP60 in activated TM cells it warrants reporting the expression of HSF1 (pS326+) in naïve cells to

show a causal relationship between HSF1 (pS326+) and HSP60 expression.
2. In Figure 2B and C, the expression of HSF1 (pS326+) and HSP60 in Tm cells in response to viral peptides does not match the corresponding flow histograms. For example, in non-stimulated cells the % of HSP60+ cells in 33.4 and 39.2% in (apparently -please label) CD4 and CD8 T cells, respectively. This does not match the graphed data. Are flow histograms available for +KR treatments?
3. On Page 10, line 221, the text indicated Hspd1 gene silencing in relations to Fig. 3 C and Fig. S2 B. However, Fig 3C Western image shows HSP60 silencing.
4. The change in nomenclature from CD4 TM to CD4 Mem could be confusing (Page 11, line 230).
5. Like Fig3C, text refers to Hspd1 gene silencing but, Fig 3d indicated HSP60 siRNA. If the authors prefer to highlight HSP60 as the gene product that may consider indicating the gene and put the protein in bracket. Alternatively, they could make clear in the text/figure legend that the gene encodes this protein and will be represented as such.
6. If HSP60 doesn't interfere with autophagy it reasoned that the glutaminase substrate glutamine may be from the exterior. It would have been interesting to measure glutamine/amino acid transporters. It would have been nice to validate the GLS and GDH1 data with additional inhibitors, especially those with clinical relevance, but additional experiments are not necessary.
7. For the seahorse data (Fig 3F) it would be good to define what KR is in the text.
8. In Line 249, page 12, "sequential addition of pharmacological agents….." The authors could expand this to add context by adding "……agents that target components of the ETC".
9. Page 12, line 253: "….CD4 TM with BPTES/R162 co-treatment and used as positive controls for reduced SRC and ATP-linked respiration (Fig. 3 A)". The figure doesn't appear to reflect this.
10. It would be useful to indicate graphically (maybe as a supplementary figure) how SRC and ATP-linked respiration are calculated. Did analysis of the raw basal OCR, ECAR or OCR/ECAR data reveal anything interesting, that may suggest whether HSP60 regulates glycolytic switch?
11. Line 267, Page 12: "HSP60 is also requested for..". Should this be "required for…."?

Version 1:

Reviewer comments:

Reviewer #1

(Remarks to the Author)
I thank the authors for properly addressing my initial comments.

Reviewer #2

(Remarks to the Author)
The authors have successfully responded to all my queries.

Open Access This Peer Review File is licensed under a Creative Commons Attribution 4.0 International License, which permits use, sharing, adaptation, distribution and reproduction in any medium or format, as long as you give appropriate credit to the original author(s) and the source, provide a link to the Creative Commons license, and indicate if changes were

made.
In cases where reviewers are anonymous, credit should be given to 'Anonymous Referee' and the source.
The images or other third party material in this Peer Review File are included in the article’s Creative Commons license, unless indicated otherwise in a credit line to the material. If material is not included in the article’s Creative Commons license and your intended use is not permitted by statutory regulation or exceeds the permitted use, you will need to obtain permission directly from the copyright holder.
To view a copy of this license, visit https://creativecommons.org/licenses/by/4.0/

Institut national de la recherche scientifique
Institut National de la Recherche Scientifique (INRS)/Institut Armand Frappier-Santé/Biotechnologie

October 6th, 2024.
Communications Biology

To Reviewers 1 and 2,

First and foremost, we thank the reviewers for their insightful and constructive comments. We also thank them for acknowledging that our study provides critical mechanistic insights onto antiviral T-cell immunity and may offer new potential ideas to therapeutically target their mitochondrial energy production. In this context, our new revised iteration includes additional data, such as (i) the ratio [glutamate]/[glutamine] in memory CD4 T cells, providing indicative values of their cellular glutamine consumption in culture, (ii) the expression levels of activated HSF-1 in naïve T-cells, (iii) representative *Seahorse* kinetic to appreciate the calculation of both SRC and ATP-linked respiration, and (iv) WB assessment of the glutamine transporter ASCT2. The new iteration further includes all technical/text edits raised by the two referees for strengthening the conclusions of our research study.
Below are our point-by-point responses to all reviewers' comments:

A. Reviewer #1:

In this manuscript, van Grevenynghe and collaborators aimed to define how mitochondrial ATP promotes the effector function of memory-phenotype CD4 and CD8 T cells, namely IL-21 production by Tfh cells and cytotoxic molecule production by CD8 T cells. Using a series of quantitative and qualitative in vitro approaches, the authors data suggest a crucial role for HSP60, a mitochondria chaperone, in the induction of glutaminolysis and FAO in CD4 and CD8 T memory cells, respectively. Overall, the study is well-developed, and the experimental design is properly described. Results offer robust evidence for the conclusions of the authors. A few additional experiments would strengthen the conclusions of this paper. More specifically:

1- If possible, the authors should track and compare, in HSP-KD versus WT cells, the glutamine consumption and incorporation into the glutaminolysis pathway, using labeled metabolomics. This would be increased evidence that engagement of glutaminolysis is indeed affected in the absence of HSP. If this experiment (as well as a similar experiment with traced fatty acids for FAO) is technically unfeasible, the authors should discuss the possible future need for such measurements.

We thank reviewer 1 for her/his valid point; Although labeled metabolomic assessments are technically unfeasible with our amounts of collected T-cells after cell transfection and our experimental platforms, we agree with referee 1 that it is still an important comment to address. Therefore, and as advised by her/him, we discuss the requirement for further metabolomic assessments in memory CD4 and CD8 T-cells in page 25, lines 543-550 of our revised manuscript (*see* section discussion). In addition, since our Bioluminescence Promega kits also provide measurements of the cellular glutamine in addition to those of glutamate, we have determined all ratio [glutamate]/[glutamine] in CD4 TM, giving us indicative values of impaired glutamine consumption when HSP60 is inhibited during cell activation (Figure S6).

531, boulevard des Prairies, Laval (Québec) H7V 1B7, Canada
Tél: (450) 687-5010 ext.4120 Fax: (450) 686-5501

2- Importantly, the only rescue experiment for HSP-KD T cells was the administration of an a-KG analog, which provides valid evidence but is not perfect: a-KG can be the byproduct of many different metabolic pathways, being a canonical intermediate of the TCA cycle. A more precise experiment would be the forced expression of glutaminolysis/FAO enzymes in cells treated with KR.

Once again, we thank referee 1 for this insightful comment, although the proposed experiment is unfeasible in our primary biological samples since it would require cell transfection with encoding plasmids. However, we acknowledge that α-KG metabolite can be the byproduct of many different metabolic pathways, and that the administration of an α-KG analog for rescuing HSP-KD in T-cells is not perfect *per se*; Therefore, we have discussed this point in our revised manuscript, and emphasized the fact that specific silencing of HSP60 or KR co-treatment impact the cellular levels of α-KG in both activated CD4 and CD8 T-cells, which, to our opinion, demonstrate the direct impact of HSP60 chaperone on proper α-KG concentrations in effector cells after TCR engagement (page 25, lines 555-558).

3- Minor issue: On line 231, please substitute "Fig. 3D" with "Fig. 3E".

It has been corrected in our revised manuscript (page 11, line 235).

B. Reviewer #2:

Through a series of metabolic inhibition and immune based assays the authors show virus specific CD4 and CD8 TM generate mitochondrial ATP through glutaminolysis and FAO pathways, and that these pathways are dependent on HSP60 induction following TCR engagement. Interestingly, they showed that while glycolysis is important early to produce inflammatory cytokines such is IFN-y, other read-outs of CD4 and CD8 TM cell effector functions such as IL-21 and granzyme B/Perforin production were reliant on HSP60-mediated OXPHOS. This work provides important mechanistic insights in T cell biology, and immunity, and offers new ideas to therapeutically modulate T cell metabolism, with potential clinical benefits. I have a few queries that are generally technical/edits.

1- Authors found increased HSF1 (pS326+) in Tm cells, which correlated with HSP60 expression. Although they found the greatest expression of HSP60 in activated TM cells it warrants reporting the expression of HSF1 (pS326+) in naïve cells to show a causal relationship between HSF1 (pS326+) and HSP60 expression.

We thank the referee 2 for her/him valid point. As such, we have also included the lower levels of activated HSF-1 pS326 in naïve CD4 and CD8 T-cells when compared to TM cells in Figure S1C of our revised manuscript (*see* page 8, lines 172-174).

2- In Figure 2B and C, the expression of HSF1 (pS326+) and HSP60 in Tm cells in response to viral peptides does not match the corresponding flow histograms. For example, in non-stimulated cells the % of HSP60+ cells in 33.4 and 39.2% in (apparently -please label) CD4 and CD8 T cells, respectively. This does not match the graphed data. Are flow histograms available for +KR treatments?

We thank the referee 2 for noticing these mistakes (aka. mislabeled histograms between non-stimulated and virus-specific T-cells). Corrections have been provided in Figures 2B and C, and representative histograms for KR treatment in virus-specific T cells have been included as well.

3- On Page 10, line 224, the text indicated Hspd1 gene silencing in relations to Fig. 3 C and Fig. S2 B. However, Fig 3C Western image shows HSP60 silencing.

To avoid any confusion, HSP60 inhibition (in bracket) have been indicated in the manuscript instead of *Hspd1* gene silencing only (page 10, line 223).

4- The change in nomenclature from CD4 TM to CD4 Mem could be confusing (Page 11, line 230).

Agree. This has been corrected (page 11, line 234 of updated manuscript).

5- Like Fig3C, text refers to Hspd1 gene silencing but, Fig 3d indicated HSP60 siRNA. If the authors prefer to highlight HSP60 as the gene product that may consider indicating the gene and put the protein in bracket. Alternatively, they could make clear in the text/figure legend that the gene encodes this protein and will be represented as such.

Same as for point 3-, HSP60 silencing (in bracket) is now indicated in the manuscript for Fig. 3D (page 11, lines 234-235).

6- If HSP60 doesn't interfere with autophagy it reasoned that the glutaminase substrate glutamine may be from the exterior. It would have been interesting to measure glutamine/amino acid transporters. It would have been nice to validate the GLS and GDH1 data with additional inhibitors, especially those with clinical relevance, but additional experiments are not necessary.

We thank the referee 2 for this point, and we have included the WB levels of the glutamine/amino acid transporter ASCT2 in CD4 TM ± HSP60 inhibition (Fig. S2B; and page 11, lines 224-226 of the revised manuscript).

7- For the seahorse data (Fig 3F) it would be good to define what KR is in the text.

Agree. This has been defined in page 12, line 265.

8- In Line 249, page 12, "sequential addition of pharmacological agents….." The authors could expand this to add context by adding "……agents that target components of the ETC".

This has been added in page 12, lines 253-254.

9- Page 12, line 253: "….CD4 TM with BPTES/R162 co-treatment and used as positive controls for reduced SRC and ATP-linked respiration (Fig. 3 A)". The figure doesn't appear to reflect this.

To avoid any confusion, we have also specified that blocking the up stream glutaminolysis pathway in CD4 TM always resulted in potent inhibition of energy production, as previously published in 2022 on Autophagy. Page 12, lines 258-260.

10- It would be useful to indicate graphically (maybe as a supplementary figure) how SRC and ATP-linked respiration are calculated.

Agree. This have been included in Figure S2E (Page 12, line 255).

11- Line 267, Page 12: "HSP60 is also requested for..". Should this be "required for…."?

This has been changed in our revised manuscript (page 13, line 275).

531, boulevard des Prairies, Laval (Québec) H7V 1B7, Canada
Tél: (450) 687-5010 ext.4120 Fax: (450) 686-5501
